# Inter-muscular networks of synchronous muscle fiber activation

**DOI:** 10.3389/fnetp.2022.1059793

**Published:** 2022-11-14

**Authors:** Sergi Garcia-Retortillo, Plamen Ch. Ivanov

**Affiliations:** ^1^ Keck Laboratory for Network Physiology, Department of Physics, Boston University, Boston, MA, United States; ^2^ Department of Health and Exercise Science, Wake Forest University, Winston-Salem, NC, United States; ^3^ Complex Systems in Sport INEFC University of Barcelona, Barcelona, Spain; ^4^ Harvard Medical School and Division of Sleep Medicine, Brigham and Women’s Hospital, Boston, MA, United States; ^5^ Institute of Biophysics and Biomedical Engineering, Bulgarian Academy of Sciences, Sofia, Bulgaria

**Keywords:** complex systems, inter-muscular coordination, muscle fibers, dynamic networks, fatigue, synchronization, network physiology

## Abstract

Skeletal muscles continuously coordinate to facilitate a wide range of movements. Muscle fiber composition and timing of activation account for distinct muscle functions and dynamics necessary to fine tune muscle coordination and generate movements. Here we address the fundamental question of how distinct muscle fiber types dynamically synchronize and integrate as a network across muscles with different functions. We uncover that physiological states are characterized by unique inter-muscular network of muscle fiber cross-frequency interactions with hierarchical organization of distinct sub-networks and modules, and a stratification profile of links strength specific for each state. We establish how this network reorganizes with transition from rest to exercise and fatigue—a complex process where network modules follow distinct phase-space trajectories reflecting their functional role in movements and adaptation to fatigue. This opens a new area of research, Network Physiology of Exercise, leading to novel network-based biomarkers of health, fitness and clinical conditions.

## 1 Introduction

The musculoskeletal system in the human organism is a complex system composed of hundreds of muscles each with diverse structure that respond individually and differently to variety of environmental influences (Schwartz, 2017). Muscles and muscle groups continuously coordinate their activation and synchronize their functions to maintain posture, stability and balance of the body at rest, to facilitate a wide range of movements, and adapt to exercise, training and fatigue. Employing reductionist approaches, investigations at the microscopic scale have predominantly focused on the structure and composition of individual muscles in relation to their specific functions through understanding the genetic composition (Wackerhage, 2014), proteomic and metabolic processes, energy exchange at the molecular and cellular level (Katch et al., 2006), neuronal activation and characteristics of individual motor units (Farina and Merletti, 2016). At the macroscopic scale, studies have traditionally focused on how individual muscles respond to external and environmental inputs, generate force, and electrophysiological patterns and dynamics across physiological states and clinical conditions related to neuro-and muscular-degeneration. However, it remains not understood how distinct type muscle fibers within individual muscles and across muscle groups dynamically synchronize their contraction activity to generate coordinated movements among muscles with different functions at the global organism level. We do not know the laws of dynamic coupling and cross-communication among muscle fibers across muscles which is essential to provide flexibility to perform a range of movements, and we do not know the basic principles of integration within modules, sub-networks and networks of inter-muscular interactions that facilitate emerging global behaviors and underlie different physiological states (rest, exercise), conditions (fatigue, training) and disease.

Inter-muscular coordination is necessary for each movement, and is associated with a specific distribution of muscle activation or force among individual muscles to produce a necessary combination of joint moments (Prilutsky, 2000). Muscular control during activities of daily living and exercise is not limited to switching muscles on or off but includes continuous fine-tuned coordination among different muscle types with precise timing and degree of activation (Kristiansen et al., 2016; Marquez et al., 2018). Such fine-tuned coordination results from the complex structure of skeletal muscles composed of various types muscle fibers that are associated with the expression of different histochemical myosin heavy chain isoforms, range from slow (oxidative) to fast (glycolytic) fibers (Schiaffino and Reggiani, 2011) and play distinct roles in generating a movement and respond differently to exercise-induced fatigue (Deshmukh et al., 2021). Distinct muscles are characterized by different composition of slow and fast muscle fibers that is essential for the specific function each muscle serves in facilitating a movement. Thus, in addition to the traditional reductionist approach with focus on individual structural components of each muscle and their dynamic characteristics, an integrative framework is needed to understand how muscle functions emerge out of coordinated interactions among muscle fibers of different type within a muscle, and how muscle fibers across multiple muscles (each with different function) integrate as a network to synchronize their activation during movement. Current investigations have not addressed the fundamental questions whether interactions between muscle fibers from different muscles are characterized by distinct coupling strength, and whether there is a unique association between network structure of muscle fiber interactions with the role of the muscles (e.g., major, supportive, compensatory) involved in a given movement, the specific physiological state (rest, exercise) and condition (levels of fatigue, training).

Traditional technologies developed to probe muscle structure and the composition of different type muscle fibers within a muscle are invasive, primarily based on biopsy, and provide a measure of the change of state for a given muscle and corresponding muscle fibers in response to exercise, fatigue and training, however, do not provide a real time information on the dynamics of muscle activity in the process of movement (Engel, 1967; Stålberg and Grimby, 1995). Surface electromyography (EMG) is non-invasive and provides information about muscle dynamics in the process of movement with high temporal resolution, however, at the integrated level of multiple motor units embedded in the muscle (Farina & Merletti, 2016). EMG signal amplitude and morphology reflect neural output from the spinal cord to the muscles, and thus, relate to the number of activated motor units with specific discharge rates (slow, intermediate, fast) and to the activation of corresponding muscle fibers within a muscle (Farina et al., 2004). Movements are produced through the control of groups of motor neurons *via* common neuronal inputs (Hug, 2011). When a group of motor neurons across several muscles receives a common input from the central nervous system, the corresponding action potentials of the different motor units occur almost simultaneously in multiple muscle areas, leading to an optimal muscle fiber contraction and precise movement (Hug, 2011; Asmussen et al., 2018). Previous studies have demonstrated that slow (type I) and fast (type II) fibers exhibit distinct spectral properties that modulate the spectral distribution of the entire muscle depending on the specific muscle fiber composition (Kupa et al., 1995; Wakeling et al., 2002; Wakeling and Rozitis, 2004; Beck et al., 2007; Wakeling, 2009; Dreibati et al., 2010; Wernbom and Aagaard, 2019; Garcia-Retortillo et al., 2020, Retortillo et al., 2021)—with frequencies below 40–60 Hz mainly attributed to the activity of small alpha motor neurons and related type I (oxidative) muscle fibers; 60–120 Hz relate to medium alpha motor neurons and type IIa fibers; and high frequencies 170–220 Hz attributed to the large alpha motor neurons and the innervated by them type IIb (glycolytic) muscle fibers (Grimby et al., 1979, 1981; Wakeling et al., 2001; Dreibati et al., 2010; Rosenblum et al., 2021).

Since changes in the spectral properties of motor units are linked to the changes in the average conduction velocity (discharge rate) of motor neurons, and the average conduction velocity of an active motor unit is related to fiber-type proportions (small diameter for type I slow fibers vs. large diameter for type II fast fibers) and fiber activation/contraction characteristics (Farina, 2008; Von Tscharner and Nigg, 2008; Casolo et al., 2022), dissecting spectral components of different frequency bands embedded in surface EMG signals is a suitable approach to infer information on motor unit recruitment and fiber-type contribution to muscle activation (Von Tscharner and Nigg, 2008; von Tscharner and Valderrabano, 2010; Casabona et al., 2021). Thus, investigations on inter-muscular interactions in the frequency domain have utilized inter-muscular coherence approaches to estimate the amount of common neural input between two muscles during voluntary motor tasks (Ushiyama and Ushiba, 2013), and to quantify the degree of shared neural inputs from cortical, subcortical and spinal influences (Grosse et al., 2002). While spectral coherence approaches have led to important findings relating activity in specific frequency bands to the role of motor cortex (Baker et al., 1997; Chang et al., 2012), impact of movement and fatigue on inter-muscular coordination among distinct muscles (Boonstra et al., 2008, Boonstra et al., 2019; Kattla and Lowery, 2010; Kerkman et al., 2018, Kerkman et al., 2020; Maillet et al., 2022; Rossato et al., 2022), or coherence modulation with maturation (Kerkman et al., 2022), coherence-based measures reflect linear aspects of interactions between same frequency band (iso-frequency coupling between muscle pairs), and cannot quantify nonlinear dynamic coupling across frequencies (Yang et al., 2018). Thus, crucial information regarding the coupling between distinct types of muscle fibers with different firing rates across muscles is ignored. Currently, we do not know the basic mechanisms underlying cross-frequency network communication of muscle fibers across muscles groups; the way muscle fibers of different type integrate their activity to facilitate fine coordination among muscles with different functions to achieve a precise movement; how this network organization relates to basic physiological states (rest, exercise), and dynamically evolves and adapts in response to exercise-induced fatigue and training. Cross-frequency coupling between different frequency bands and continuous dynamic exchange of information within a network of frequency bands may be the carrier mechanism for integrating local and global processes essential to facilitate flexibility to inputs and to generate a rich phase space of behaviors and states in a range of physiological systems (Jirsa and Müller, 2013; Liu et al., 2015; Lin et al., 2016, Lin et al., 2020; Rizzo et al., 2020; Chen et al., 2022). This underlines the necessity to go beyond traditional approaches and explore muscle function and muscle coordination through networks of muscle fiber interaction.

Here we apply a Network Physiology framework (Bashan et al., 2012; Ivanov and Bartsch, 2014; Bartsch et al., 2015; Ivanov et al., 2016; Ivanov, 2021) to explore how muscle fibers from different muscles synchronize their activity during movements. We develop a new analytical approach and experimental protocol where cross-frequency coupling across muscle groups is studied during rest and repeated periods of prolonged maximal exercise (Section 2.2, Methods). The neuro-muscular system presents a complex network organization of muscle groups and muscle fibers which dynamically interact in a non-linear way to coordinate functions and generate movements. Recent studies have demonstrated that skeletal muscles are characterized by 1) spectral power profiles that are specific for different-type muscles, and 2) the different frequency bands within the spectral profile of a given muscle (which are associated with the activity of distinct muscle fiber types) exhibit a differentiated response during different physiologic states (rest, exercise) and with accumulation of fatigue (Garcia-Retortillo et al., 2020; Garcia-Retortillo et al., 2021). This raises the hypothesis that interaction among muscle fibers and coupling between their respective frequency bands is essential for generating synchronized activation across muscles with different functions to support the body at rest and to facilitate coordinated movements. Coordinated network interactions of muscle fibers across muscles could be achieved through several potential principles of network integration where 1) interactions among muscle fibers within a given muscle vertically integrate into global output muscle dynamics which synchronize with the global dynamics from another muscle; 2) muscle fibers of a given type within a muscle synchronize with the same type fibers from another muscle through horizontal network; or alternatively 3) all types muscle fibers of a given muscle synchronize to various degree of coupling their frequency bands activity with all fibers from another muscle, leading to a scenario with highest degree of complexity in network interactions to account for maximum precision of synchronization among distinct muscles and to maximum flexibility in response to various tasks, exercise demands and levels of fatigue. We also hypothesize that muscle fiber network interactions exhibit specific hierarchical organization of coupling (network links) strength for each pair of muscles involved in a given movement depending on the muscle type and function (e.g., major, supportive, compensatory) in the context of the movement, and thus, different network structure and dynamics of muscle fiber interactions would characterize distinct physiological states (rest, exercise). Further, we hypothesize that the degree of coupling (synchronous activation) among muscle fibers across muscles changes with accumulation of fatigue, and exhibits differentiated response depending on the muscle fiber type (frequency bands) and muscle function, thus, leading to hierarchical reorganization of the network.

Given that coordination between trunk and leg muscles is required for variety of functional tasks, there is a need to quantify the complex dynamics underlying muscle fiber network interactions between these muscles, and how their coordination changes over prolonged periods of extended and repeated exercises. To this end, we use a protocol including repeated long bouts of squat exercise performed until exhaustion (Garcia-Retortillo et al., 2020). The squat test can be considered as an administrable and reliable tool to assess coordination among different muscle fibers across muscles groups (Bianco et al., 2015; McAllister and Costigan, 2019). We analyze EMG data from muscles with high myoelectrical activity during the squat movement, but with distinct histochemical composition and different levels of activation and contribution to the exercise effort (Khaiyat and Norris, 2018): 1) erector spinae back muscle composed predominantly of type I muscle fibers (Cagnie et al., 2015) and 2) vastus lateralis leg muscle with higher percentage of type II muscle fibers (Vromans and Faghri, 2018; Nederveen et al., 2020).

We address the fundamental question of how distinct muscle fiber types dynamically synchronize with each other and integrate as a network across back and leg muscles during rest and exercise, and how the network of muscle fiber interactions reorganizes with accumulation of fatigue. We uncover that each physiologic state (rest, exercise) is characterized by a unique inter-muscular network organization with state-specific patterns in network links strength stratification reflecting complex cross-frequency communication among distinct types of muscle fibers across muscles, and that inter-muscular coupling strength between muscle fibers dramatically increases with transition from rest to exercise to coordinate force generation under higher physiological demand. Our analyses show that the inter-muscular network of muscle fiber interactions exhibits a hierarchical structure of sub-networks and modules with distinct profiles of links strength stratification depending on the type of muscle fiber—each muscle pair presents specific signature of cross-frequency communication to synchronize activation among distinct muscle fibers types, which depend on muscle histochemical characteristics and on the role each pair of muscle plays (e.g., major, supportive, compensatory) during squat movements. Further, we uncover that network structure and inter-muscular interaction profiles among muscle fibers dynamically reorganize with accumulation of fatigue, where different sub-networks and modules corresponding to different pairs of muscles exhibit a differentiated response of decrease or increase in the degree of coupling (network links strength) among muscle fibers. Notably, the proposed here dynamic network approach allows to track how coordination between muscle pairs in the process of movement gradually evolves with exercise and progression of fatigue, and to establish a novel phase diagram representing the relative contributions of each pair of muscles based on their role in the movement. The reported empirical findings break new ground in the study of inter-muscular interactions through detailed quantification of the role different muscle fibers play in generating muscle coordination, provide new understanding of the basic physiological mechanisms of muscle fiber network integration, and can help derive novel network-based measures to assess effects of sports performance, fatigue, overtraining or muscle-skeletal injuries.

## 2 Methods

### 2.1 Subjects

Fourteen healthy young adults (six males and eight females; age 22.19 ± 3.56 years, height 174.69 ± 10 cm, and mean body mass 66.81 ± 13.39 kg) were recruited from a larger pool of undergraduate students from the University of Girona, Spain. With the aim of ensuring a homogenous sample, participants were strictly recruited according to the following inclusion criteria: 1) aged 20–30 years; 2) BMI (in kg/m^2^) > 18.5 and <30; 3) normal physical activity >5 and <10 h/week, but without sport specialization (not active athletes), and 4) blood pressure <140/90 mmHg. The following exclusion criteria were applied: 1) intake of prescribed drugs that could negatively affect muscle strength, such as corticosteroids; 2) no current or previous injury that could prevent performance during the experimental protocol test, and 3) any other physical condition (cardiac, respiratory etc.) that may have prevented the performance of a test protocol involving squat exercise until exhaustion. The experimental protocol was approved by the local ethical committee, and was carried out according to the Helsinki Declaration. Before taking part in the study, participants read the study description and risks, and signed an informed consent.

### 2.2 Study design and test protocol

**FIGURE 1:**
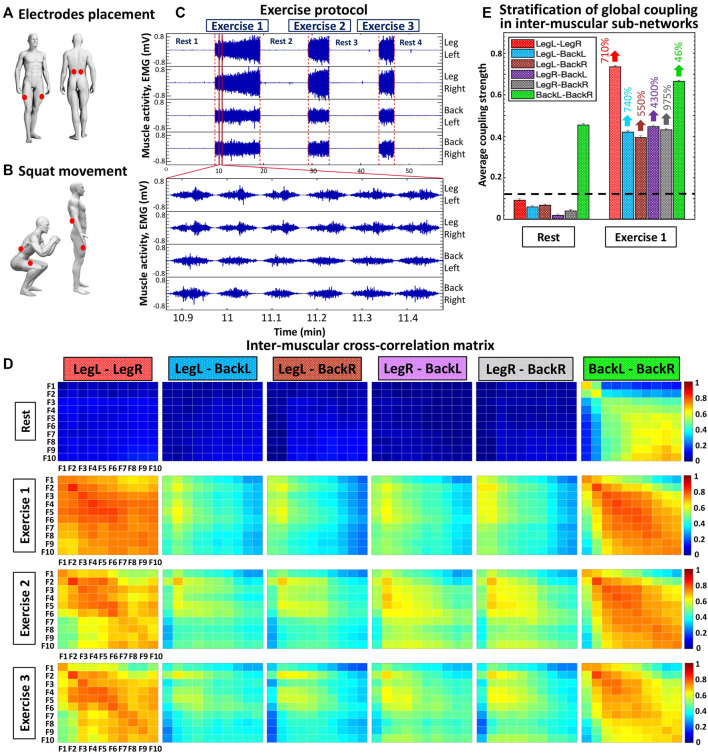
Experimental set up, exercise protocol and cross-correlation matrices representing networks of inter-muscular interactions among rhythms corresponding to the activation of different muscle fibers during rest and exercise. **(A)** EMG electrodes placement (red dots) of simultaneously recorded muscle groups: left and right vastus lateralis (LegL and LegR muscles); left and right erector spinae (BackL and BackR lower back muscles). **(B)** Schematic representation of squat movement that requires continuously coordinated activity between lower back and leg muscles. **(C)** EMG signals from a typical healthy subject represent the evolution of myoelectrical activity for each muscle group shown in **(A)** during rest and repeated exercise bouts consisting of consecutive squat movements, where the EMG amplitude increases with progression of fatigue during the study test protocol (Section 2.2, Methods). The exercise protocol comprises three consecutive squat bouts (Exercise 1, 2 and 3), each performed until exhaustion, separated by 10-min rest periods in supine position. **(D)** Inter-muscular cross-correlation matrices where matrix elements represent the group average coupling (degree of synchronization) between rhythms of myoelectrical activation corresponding to the activity of different muscle fibers for all pairs of muscle groups (LegL-LegR, LegL-BackL, LegL-BackR, LegR-BackL, LegR-BackR, BackL-BackR) during rest and exercise bouts. Each matrix represents a sub-network of inter-muscular interactions with complex organization of network links (coupling strength) among myoelectrical rhythms (10 frequency bands F1, F2, … , F10 with equal width of 19.5 Hz in the intervals (5–45 Hz) and (55–215 Hz); see Methods) for each pair of muscle groups (matrix rows correspond to the first muscle group and matrix columns to the second muscle group in the pair; color code in vertical bars indicates coupling strength). **(E)** Stratification in the average coupling strength for each inter-muscular sub-network during Rest 1 and Exercise 1 — bars represent the matrix elements average value for each matrix shown in **(D)**. Error bars on top of each bar indicate the standard error; horizontal black dotted line marks the threshold of physiological significance for network interactions of myoelectrical rhythms (muscle fibers) across muscle groups based on a surrogate test where rhythms from different subjects are randomly coupled (Section 2.6, Methods).

In the utilized experimental protocol (schematic diagram shown in Figure 1C) participants visited the laboratory for two sessions, separated by a 2-day interval. During the first session (i.e., familiarization), participants practiced the squat test until they were able to execute the movement according to protocol (see study test protocol below). In the second session, participants performed the study test protocol, and physiological data were recorded.

The protocol consists of consecutive rest and exercise segments: 1) 10-min rest period in supine position (Rest 1); 2) squat test performed until exhaustion (Exercise 1); 3) 10-min rest in supine position (Rest 2); 4) squat test performed until exhaustion (Exercise 2); 5) 10-min rest period in supine position (Rest 3); 6) squat test performed until exhaustion (Exercise 3), and 7) 10-min rest period in supine position (Rest 4).

Rest segments. During Rest 1, 2, 3, and 4 periods participants laid down in a supine position on a massage table. With the aim to avoid joint compression and facilitate relaxation, a pillow was placed under the participants’ knees. A second pillow was placed under the participants’ back to avoid contact between the back electrodes and the table.

Exercise segments. During Exercise 1, 2, and 3 segments participants performed a squat test (Figure 1B) until exhaustion according to the following instructions: 1) feet placed apart a bit wider than shoulder-width; 2) arms extended out straight; 3) movement initiation by inhaling and unlocking the hips, slightly bringing them back; 4) keep sending hips backward as the knees begin to flex; 5) squat down until touching a guiding rope designed to secure that all participants perform the same range of movement (see details below); 6) return to standing position, and 7) repeat the squat movement until exhaustion. The guiding rope was placed at a height corresponding to the level of participants’ thighs at the bottom of the squat movement when thighs are parallel to the ground. Participants were instructed to keep their chest up, weight over the heels, and not to allow knees falling into a valgus position (Bianco et al., 2015). Given that the back squat is more commonly used in training and every day movement compared to the front squat variation (Yavuz et al., 2015), and since the front squat requires higher ankle mobility (loss of ankle dorsiflexion is a common feature in general population) (Rabin and Kozol, 2017), the back squat was selected for the exercise protocol in the current study. The squatting pace was controlled by a metronome (MetroTimer version 3.3.2, ONYX Apps), using a 3:3 tempo with 3 s down movement and 3 s up. Thus, one single squat lasts 6 s. Since in our protocol squat movements during each exercise segment were performed till exhaustion, each squat exercise segment finished when participants were not able to do the next squat down/up movement or, alternatively, when they could not maintain the required 3:3 squat tempo.

The repetition of three consecutive squat exercise segments performed until exhaustion, allows to identify the effect of accumulated fatigue on Leg and Back muscles, and track the evolution of muscle network interactions across exercise segments.

### 2.3 Electromyography acquisition and EMG signal processing

Participants were asked to wear appropriate clothing that would not obstruct EMG electrode placement sites. Before the mounting of the EMG electrodes, participants’ skin was shaved and cleaned using alcohol, and left to dry for 60 s to reduce the myoelectrical impedance, according to the SENIAM guidelines (Hermens, 2000). EMG signals from the following muscles were recorded simultaneously during the entire exercise protocol: left and right vastus lateralis (Leg-Left and Leg-Right muscles); left and right erector spinae longissimus (Back-Left and Back-Right lower back muscles). The exact location of the surface electrodes (Ag/AgCl bipolar surface electrodes, Sorimex, Toruń, Poland) placement on each muscle group was also carried out according to the recommendations of SENIAM organization. More specifically, vastus lateralis electrodes were placed at 2/3 on the line from the anterior spina iliaca superior to the lateral side of the patella, and the erector spinae electrodes were located at a 2-finger width lateral from the spinous process of vertebra L1 (Figure 1A). After the electrodes were secured, a quality check was performed to ensure EMG signal validity. The aforementioned Leg and Back muscles were selected since they present the highest myoelectrical activity during bodyweight squat movement (Khaiyat and Norris, 2018).

Data were recorded using Biopac MP36 (Biopac Systems Inc., Goleta, CA, United States) and processed by means of Matlab (Mathworks, Natik, MA, United States). Raw data was recorded at a sample frequency of 500 Hz and filtered online using a 5–250 Hz band-pass filter. A Notch filter was used with a width of 1 Hz at the frequency of 50 Hz (i.e., 49.5–50.5 Hz) to remove line interference from the grid. All EMG recordings were visually inspected and only signals without noise artifacts and missing data were utilized in the analysis.

### 2.4 Spectral decomposition

To uncover how distinct muscle fibers dynamically coordinate and synchronize their activation and integrate as a network across different muscle groups to generate optimal function during the squat movement, we first segment the EMG signals from the left and right vastus lateralis (Leg-Left and Leg-Right muscles) and the left and right erector spinae longissimus (Back-Left and Back-Right lower back muscles) into 2-s non-overlapping time windows across consecutive Rest and Exercise periods. Within each 2-s time window, we extract the spectral power S(f) from each EMG signal using the “pwelch” function in Matlab, based on the discrete Fourier transform (DTF) and the Welch’s overlapped segment averaging estimator. For each time window we obtain a spectral power value in bins of 0.5 Hz for the range (5–250 Hz), that is, *N* = 500 is the number of spectral power data points for each window of 2 s. To probe specific contributions from different frequency bands F_i_ to the spectral power within each 2-s time window of the EMG signal, we consider 10 frequency bands with equal width of 19.5 Hz. These frequency bands correspond to the range of activity of different types of muscle fibers in each Leg and Back Muscle, i.e., F1 = (5–24.5 Hz), F2 = (25–44.5 Hz), F3 = (55–74.5 Hz), F4 = (75–94.5 Hz), F5 = (95–114.5 Hz), F6 = (115–134.5 Hz), F7 = (135–154.5 Hz), F8 = (155–174.5 Hz), F9 = (175–194.5 Hz) and F10 = (195–214.5 Hz).

We calculate the sum of the power $\tilde{S}\left(f\right)$ across all frequency bins within each frequency band: $\tilde{S}\left(f\right):=\overset{n}{\underset{i=1}{\sum }}S\left(f_{i}\right)$ , where $f_{i}$ are all n = 39 frequencies considered in each frequency band F_i_. Thus, we obtain 10 time series of EMG band power $\tilde{S}\left(f\right)$ with 2-s resolution for each muscle during rest and exercise protocol segments, representing the dynamics of all representative EMG frequency bands. Frequencies below 40–60 Hz (corresponding to our frequency bands F1, F2 and F3) are attributed to the activity of small alpha-motor neurons that innervate type I slow muscle fibers. The frequency range 60–120 Hz (bands F4, F5, F6 and F7) are attributed to medium alpha-motor neurons that innervate type IIa intermediate (oxidative) fibers, and high frequencies in the range 170–220 Hz (bands F8, F9 and F10), correspond to large alpha-motor neurons that connect to type IIb fast (glycolytic) fibers (Grimby, Hannerz, & Hedman, 1979; Grimby, Hannerz, & Hedman, 1981; Wakeling, Pascual, Nigg, & Von Tscharner, 2001; Dreibati et al., 2010). The (45–54.5 Hz) range is filtered out by the Notch filter centered at 50 Hz to remove interference from electric grid, which modifies the EMG signal altering the spectral power of frequencies around 50 Hz. The obtained ten times series of EMG spectral power for each band F_i_ are then normalized to zero mean (µ = 0) and unit standard deviation (*σ* = 1). The obtained time series of EMG power $\tilde{S}\left(f\right)$ in each frequency band F_i_ capture not only the quasi-stationary behavior of distinct EMG frequency bands during a specific physiological state (i.e., high or low EMG amplitude corresponding to a given state) but also reflects the micro-architecture (in 2-s resolution) of synchronous modulation in the amplitude of muscle activation within a given physiological state that gives rise to effective couplings, and allows to track variations in coupling and network interactions of EMG frequency bands F_i_ (corresponding to muscle fibers with distinct function) across different muscle groups with transitions from Rest to Exercise and with accumulation of fatigue for repeated exercise bouts (Figure 1C).

### 2.5 Cross-correlations between time series of EMG spectral power in different frequency bands

**FIGURE 2:**
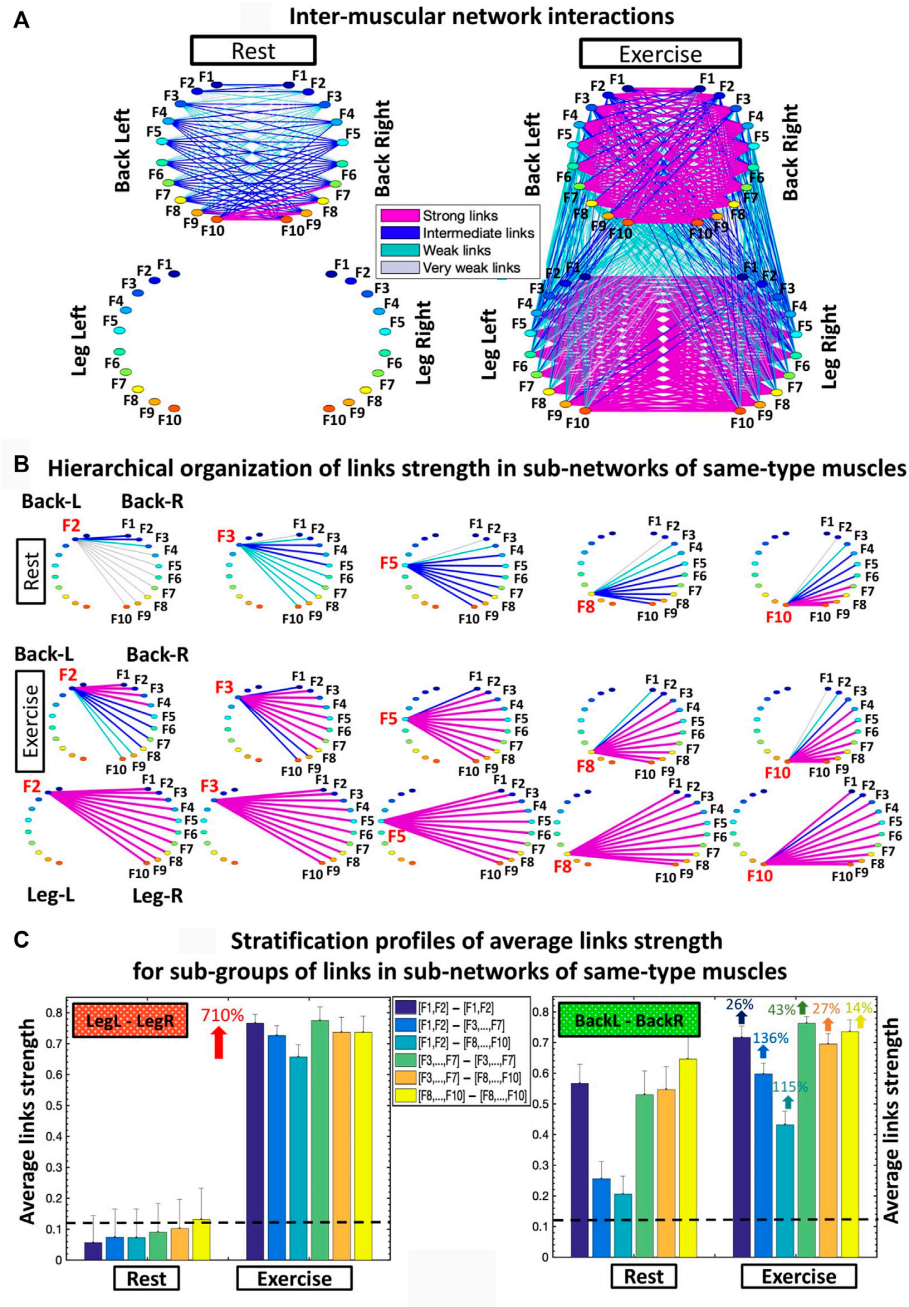
Networks of interactions among muscle fibers across muscle groups. **(A)** Dynamic networks of inter-muscular interactions between the four major muscle groups involved in squat movement show markedly distinct organization during Rest and Exercise. Network maps are obtained based on the group-averaged cross-correlation matrices for the Rest 1 and Exercise 1 periods shown in Figure 1D. Network links correspond to the matrix elements and represent the coupling strength between rhythms of myoelectrical activation (frequency bands F_*i*_), quantifying the degree of synchronous activity of muscle fibers from different muscle groups. Inter-muscular network interactions are heterogeneous with hierarchical organization of link strength within sub-networks for pairs of muscle group. Each muscle is shown as a semi-circle, where color nodes represent frequency bands (Section 2.4, Methods) associated with the activity of distinct muscle fiber types within each muscle group. Links strength is marked by line color and width (Section 2.8, Methods). **(B)** Hierarchical structure of links strength for the same-type muscle LegL-LegR and BackL-BackR sub-networks during Rest and Exercise. Shown are only the sub-network modules for low (F2, F3), intermediate (F5) and high (F8, F10) frequency bands extracted from the network maps in **(A)** using the same color code. **(C)** Stratification profiles of links strength for the same-type muscle LegL-LegR and BackL-BackR sub-networks during Rest and Exercise, shown by six bars corresponding to the group-average links strength of all muscle fiber interaction modules in each sub-network. Error bars indicate the standard error. Black dashed line marks the threshold of physiological significance for network links strength obtained from a surrogate test where frequency bands from different subjects are paired (Section 2.6, Methods).

To investigate cross-frequency interactions among muscle fibers (corresponding to EMG frequency bands) that occur as a result of synchronous modulation of their spectral amplitudes at short timescales on top of large scale quasi-steady state behavior associated with specific physiologic states, we consider all pairs of major muscle groups involved in squat movement: 1) interactions of all frequency bands from same-type muscles (LegL-LegR and BackL-BackR) and 2) different-type muscles (LegL-BackL, LegL-BackR, LegR-Back and LegR-BackR). For each protocol segment (Rest, Exercise 1, Exercise 2 and Exercise 3) and for each muscle pair, we calculate the bivariate equal-time Pearson’s cross-correlation for all pairs of time series representing EMG spectral power $\tilde{S}\left(f\right)$ in the frequency bands F_i_ where i = 1, … ,10. This leads to 10 × 10 = 100 cross-correlation values C_i,j_ for each pair of muscles, as shown in the inter-muscular cross-correlation matrices (Figure 1D)—i.e., C_i,j_ quantifies the degree of coupling EMG frequency band F_i_ from one muscle with the frequency band F_j_ from another muscle. The cross-correlation values range from C_i,j_ = −1 (fully anti-correlated) to C_i,j_ = 1 (fully positively correlated), with C_i,j_ = 0 indicating the absence of linear relation between the power $\tilde{S}\left(f\right)$ time series of two EMG frequency bands. Networks representing inter-muscular interactions are derived from the cross-correlation matrices in Figure 1D, where the matrix for a given pair of muscles at a given state (Rest or Exercise) corresponds to a sub-network with network links representing the degree coupling (synchronous activation) of distinct muscle fibers (frequency bands) from the two muscles. A multiplex network of sub-networks is obtained to visualize interactions among muscle fibers from all pairs of muscle groups and their hierarchical organization within the network, where higher cross-correlation values correspond to stronger network links (Figure 2A).

### 2.6 Fourier phase randomization surrogate test and significance threshold for links strength in networks of inter-muscular interactions

To demonstrate the physiological significance of the networks of inter-muscular interactions obtained from our empirical analysis, we perform a Fourier phase randomization surrogate test on the EMG signals recorded from the four major muscle groups (LegL, LegR, BackL and BackR) during squat movement. The test preserves the global spectral power in the different frequency bands F_i_ within the EMG recording but destroys Fourier phase information related to nonlinear EMG characteristics. Thus, the test eliminates heterogeneities in the fine temporal structure associated with short time scale modulations in the spectral dynamics of EMG frequency bands F_i_ within a muscle, which reflect activation patterns of different muscle fiber types. Note that in the Fourier-phase-randomized surrogate EMG signals the relative ratios among the average spectral power of muscle activation in the frequency bands F_i_ are preserved, while synchronous modulations in EMG frequency bands that underlie effective cross-frequency coupling and account for the nonlinear characteristics of EMG signals are eliminated. As a result, the degree of coupling between EMG frequency bands F_i_ from different muscles is significantly reduced after the Fourier Phase Randomization procedure, since physiologically relevant information regarding coordinated and synchronous activation of distinct type muscle fibers from different muscles is lost.

**FIGURE 3:**
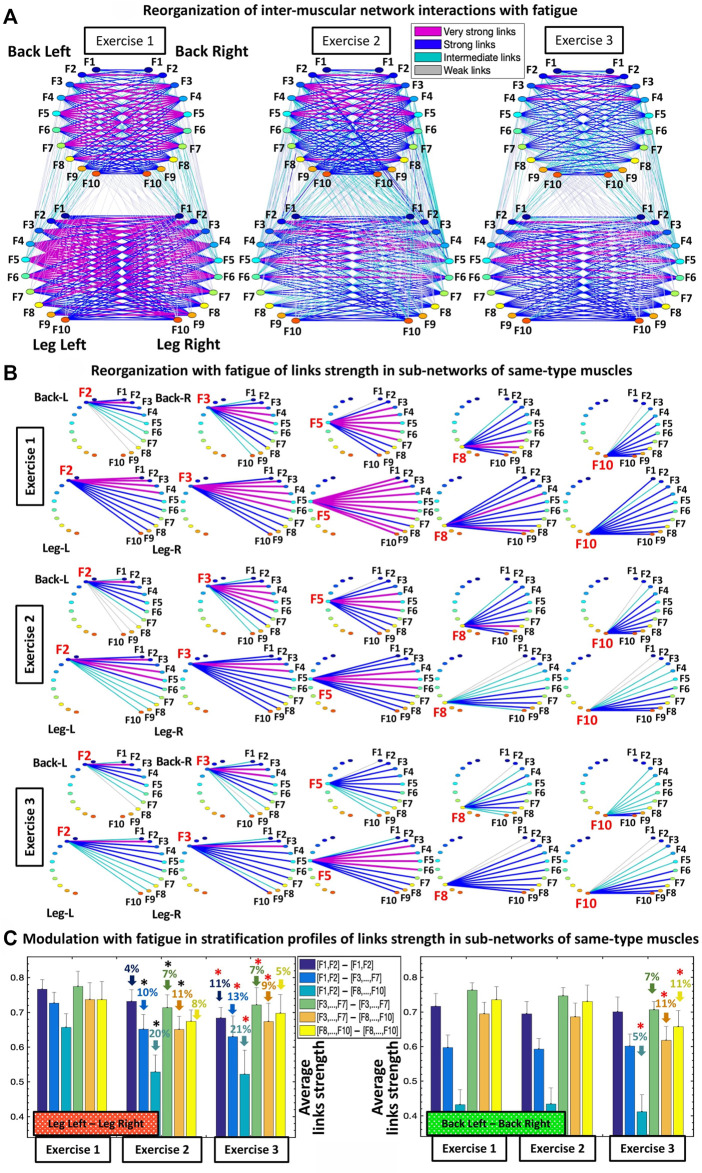
Networks of interactions among muscle fibers across muscle groups and their reorganization with fatigue. **(A)** Dynamic networks of inter-muscular interactions between major muscle groups involved in squat movement show network evolution for repeated bouts of exercise and reorganization with accumulation of fatigue. Network maps are obtained based on the group-averaged cross-correlation matrices for Exercise 1, 2, and 3 shown in Figure 1D, where network links correspond to the cross-correlation matrix elements and represent the coupling strength between rhythms of myoelectrical activation (frequency bands) corresponding to the degree of synchronous activity of muscle fibers from different muscle groups. Inter-muscular interactions form a multiplex network with marked heterogeneity characterized by different topology and hierarchical organization of links strength for sub-networks representing pairs of same- and different-type muscle groups. Muscle groups are shown as semicircles where color nodes represent distinct frequency bands (Section 2.4, Methods) associated with the activity of distinct muscle fiber types within each muscle group. Links strength is shown by line color and width (Section 2.8, Methods). **(B)** Hierarchical structure of links strength for the same-type muscle LegL-LegR and BackL-BackR sub-networks during Exercise 1 which play main role in squat movement, and their reorganization with accumulation of fatigue during Exercise 2 and 3. Shown are as examples the sub-network modules for low (F2, F3), intermediate (F5) and high (F8, F10) frequency bands extracted from the network maps in **(A)** using the same color code. **(C)** Stratification profiles of links strength for the same-type muscle LegL-LegR and BackL-BackR sub-networks and profile reorganization for consecutive exercise bouts in response to accumulation of fatigue. Links strength stratification profiles for the LegL-LegR and BackL-BackR sub-networks are shown by six bars corresponding to the group-average links strength of all muscle fiber interaction modules in each sub-network. Error bars indicate standard error. Black and red stars indicate statistically significant differences in links strength for all modules comparing Exercise 2 vs. Exercise 1, and Exercise 3 vs. Exercise 1, respectively (Wilcoxon test *p* values <0.04).

**FIGURE 4:**
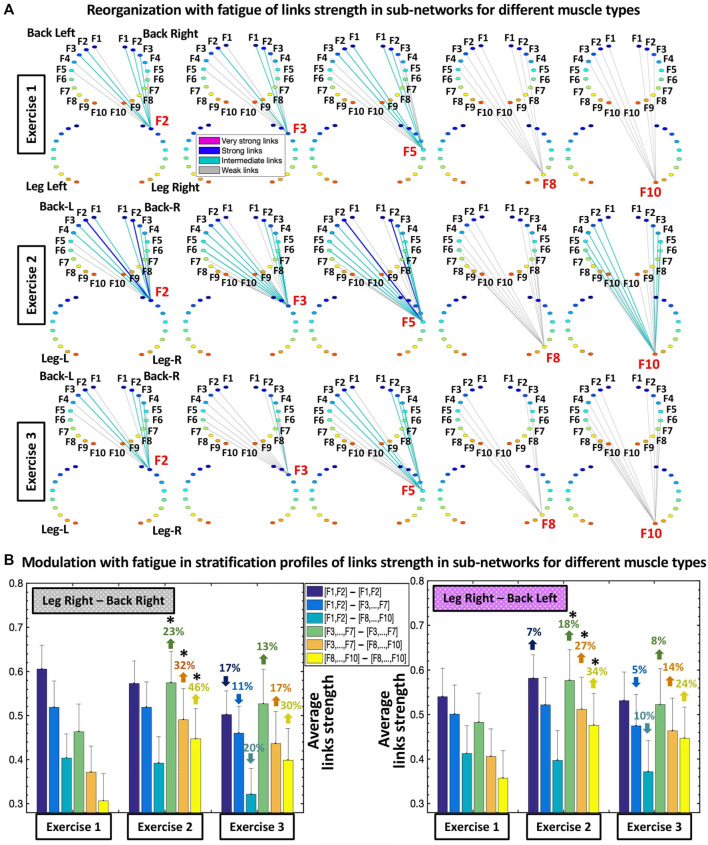
Sub-networks representing synchronous activity of muscle fibers for pairs of different muscle types and network evolution for repeated bouts of exercise. **(A)** Hierarchical organization of links strength in two sub-networks showing muscle fiber interactions between different types of muscles LegR-BackR and LegR-BackL, which play supportive/compensatory role in squat movement. Shown are examples of sub-network modules for low (F2, F3), intermediate (F5) and high (F8, F10) frequency bands (representing the activity of different muscle fibers within each muscle group) extracted from the network maps in Figure 3A using the same color code. A similar network structure and reorganization across exercise bouts is observed for the other two sub-networks LegL-BackL and LegL-BackR of different muscle types (not shown). **(B)** Stratification profiles of links strength for the different-type muscles LegR-BackR and LegR-BackL sub-networks and profile reorganization for consecutive exercise bouts in response to accumulation of fatigue. Stratification profiles are shown by six bars corresponding to the group-average links strength for all muscle fiber interaction modules in each sub-network. Error bars indicate standard error. Black stars indicate statistically significant differences in links strength for all modules comparing Exercise 2 vs. Exercise 1 (Wilcoxon test *p* values <0.05).

**FIGURE 5:**
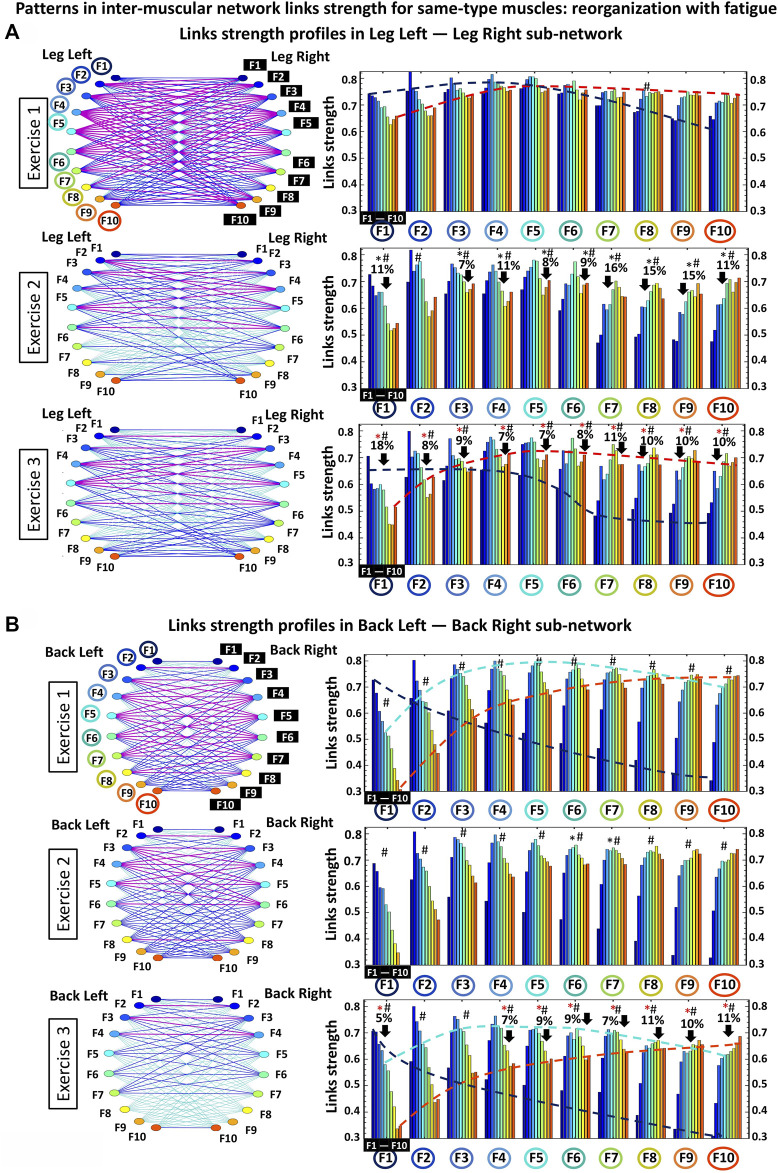
Hierarchical organization of network modules and their links strength profiles within same-type muscle sub-networks. **(A)** Dynamic networks of interactions among muscle fibers (represented by different frequency bands Fi) within the same-type muscle LegL-LegR sub-network and reorganization of links strength for the basic modules in the sub-network with repeated exercise bouts (left panels). Networks represent the group-averaged cross-correlation matrices for Exercise 1, 2 and 3 in Figure 1D, where network links correspond to the matrix elements and show the coupling strength (degree of synchronous activity) of muscle fibers from the LegL and LegR muscles. Links strength is marked by line color and width (Section 2.8, Methods). The LegL-LegR sub-network topology is defined by basic modules, each representing the interaction of a given frequency band from one muscle (LegL) with all frequency bands from the other muscle (LegR) — i.e., ten sub-network modules with ten links within each module form a complex hierarchical organization of links strength in the LegL-LegR sub-network modulated by accumulation of fatigue with repeated exercise. Frequency bands F_i_ of the LegL muscle are marked by circles on the horizontal axis of each panel, and frequency bands F_i_ of the LegR muscle are marked by black squares within each module. Bars color in each profile corresponds to the color of the node associated with a given frequency band in the Leg muscle. Black and red stars indicate statistically significant differences in links strength for all modules comparing Exercise 1 vs. Exercise 2, and Exercise 1 vs. Exercise 3, respectively (Wilcoxon test *p* values <0.04; Hashtags show significant stratification of links strength within a module (Friedman ANOVA *p* values <0.03) Dash lines with different color mark the modulation of the profiles. **(B)** Same network representation and links strength profiles of sub-network modules as in **(A)** are shown for the same-type muscle BackL-BackR sub-network. Notably, modules in the BackL-BackR sub-network exhibit a similar shape for their links strength profiles as modules in the LegL-LegR sub-network, however, with significantly higher stratification of links strength within each module as well as less pronounced decline in links strength (only during Exercise 3) and preserved degree of links strength stratification within profiles for all exercise bouts.

**FIGURE 6:**
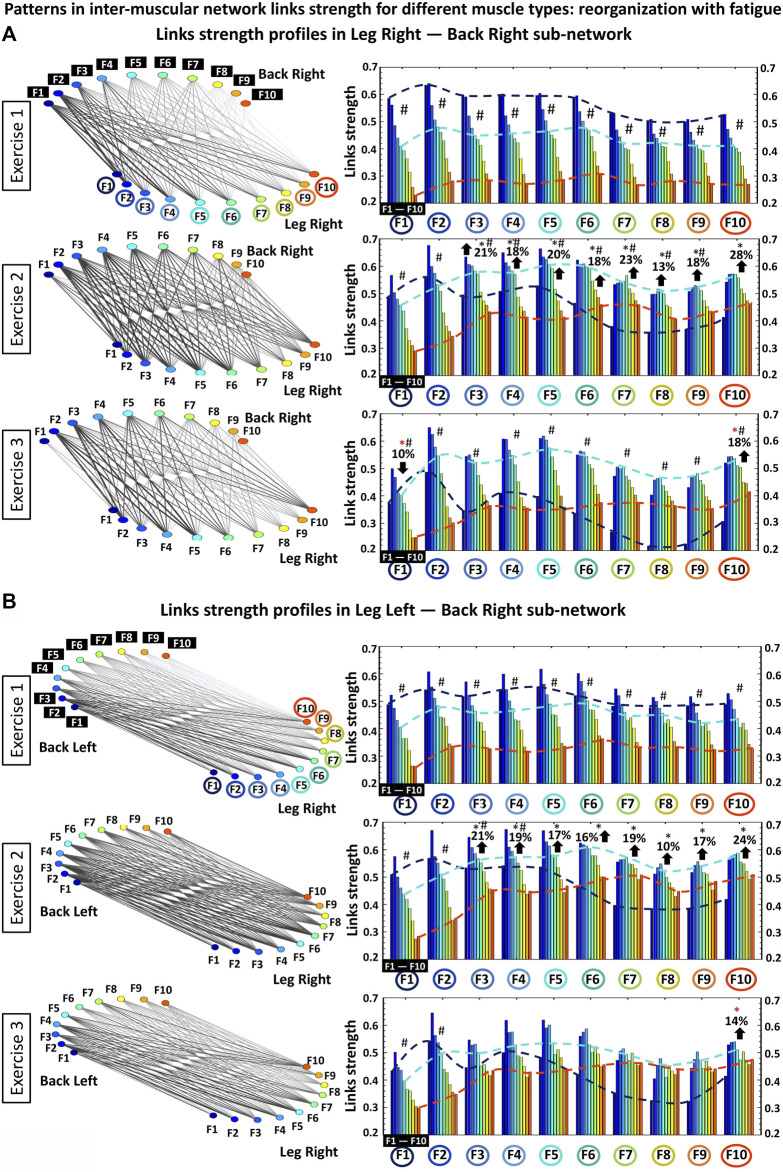
Profiles of links strength and hierarchical organization of links in modules within different-type muscle sub-networks. **(A)** Networks of interaction among muscle fibers within the different-type muscle LegR-BackR sub-network where links strength represents the degree of synchronous activation in the spectral power of muscle fibers working in different frequency ranges (frequency bands F_i_) within each muscle group. During exercise the LegR-BackR sub-network topology is characterized by a hierarchical organization of basic modules, where each module represents the interaction of a given muscle fiber type (frequency band) from one muscle (LegR) with all muscle fibers (frequency bands) from the other muscle (BackR) in the sub-network. Frequency bands F_i_ of the LegR muscle are marked by circles on the horizontal axis of each panel, and frequency bands F_i_ of the BackR muscle are marked by black squares within each module. Bars color in each profile corresponds to the color of the node associated with a given frequency band in the muscle. Black and red stars indicate statistically significant differences in links strength for all modules comparing Exercise 1 vs. Exercise 2, and Exercise 1 vs. Exercise 3, respectively (Wilcoxon test *p* values <0.04); Hashtags show significant stratification of links strength within a module during a given exercise bout (Friedman ANOVA *p* values <0.02). Dash lines with different color mark the modulation of the profiles. **(B)** Same network representation and links strength profiles of sub-network modules as in **(A)** are shown for the different-type muscle LegR-BackL sub-network. Notably, modules in the LegR-BackL sub-network exhibit 1) similar shape for their links strength profiles as the modules in the LegR-BackR sub-network; 2) similar increase of the average link strength during Exercise 2; and 3) similar modulation of the profiles shape during Exercise 2 and 3 in response to fatigue.

Further, to test the statistical significance and physiological relevance of the observed hierarchical network organization of inter-muscular interactions (Figures 2–4), the specific profiles of links strength within sub-networks representing different muscle pairs and their reorganization with fatigue (Figures 5, 6), we perform an additional step in our surrogate test to establish the significance threshold for network links strength. Specifically, for each network link, surrogates are generated considering signals from each combination of two randomly chosen subjects. Since we have 14 subjects in the database, 91 pairs of random subject combinations are generated. Each muscle pair involves 100 links within the corresponding sub-network between 10 frequency bands F_i_ (muscle fibers) of each muscle. Thus, combining the six sub-networks representing all muscle pairs we obtain a distribution of 54,600 surrogate links (cross-correlation values) for each protocol segment (Rest, Exercise 1, Exercise 2 and Exercise 3)— i.e., 6 muscle pairs x 100 links x 91 subject combinations = 54,600 surrogate links. For each protocol segment distribution the mean µ_*surr*_ and standard deviation σ_*surr*_ are obtained. Thus, the significance threshold at 95% confidence level for the network links strength is defined as µ_*surr*_ + 2*σ*_*surr*_ for each protocol segment. We find that the significance threshold for network links strength during Rest is Th_rest_ = 0.120 and during Exercise is Th_exercise_ = 0.116 (corresponding to the highest Th value during the exercise segments. The physiological significance threshold is represented by horizontal dashed black lines in all figure panels showing bar plots of group average links strength within sub-networks, network modules (Figures 2–4) and profiles of individual links strength (Figures 5, 6).

### 2.7 Inter-muscular cross-correlation matrices

Group-averaged cross-correlation matrices represent pairwise coupling strength between the ten frequency bands F_i_ of one muscle with the same bands derived from another muscle (i.e., 6 distinct muscle pairs: LegL-LegR, LegL-BackL, LegL-BackR, LegR-BackL, LegR-BackR and BackL-BackR) during a given protocol segment (Rest, Exercise 1, Exercise 2 and Exercise 3) (Figure 1D). Matrix elements indicate the group-averaged (for all 14 subjects in the database) coupling strength between a frequency band that represents the activation of a specific muscle-fiber type in a given muscle and a frequency band from another muscle. The dynamics of each muscle is represented by 10 EMG frequency bands with equal width of 19.5 Hz in the interval (5–215 Hz) (Section 2.4, Methods), and thus, each matrix consists of 100 elements (cross-correlation coefficients) representing muscle fiber interactions for each pair of muscles at a given physiological state (Rest or Exercise; Figure 1D).

### 2.8 Inter-muscular interaction networks

To visualize the information provided by the inter-muscular cross-correlation matrices, we map the group-averaged matrices in Figure 1D into different networks for the Rest, Exercise 1, Exercise 2 and Exercise 3 segments of our protocol. The graphical approach we employ is essential to identify universal patterns in the inter-muscular network structure, the hierarchical organization of sub-networks and modules, and to track the transition in network characteristics across different physiological states. Each muscle is represented by a semicircle where color nodes represent distinct frequency bands F_i_ corresponding to different muscle fiber types in the muscle (Section 2.4, Methods). Network links correspond to the values of cross-correlation matrix elements C_i,j_ in Figure 1D and reflect the coupling strength between the frequency bands from two different muscles, where the frequency bands nodes and links for each pair of muscles form a separate sub-network (Figures 2, 3). Links strength is marked by line color and width–we use distinct link color-code to demonstrate the contrast in network structure between physiological states and network reorganization with fatigue. To illustrate the differences in network organization for Rest vs. Exercise (Figure 2) we use the following links strength classification: weak links (0.2 < C_i,j_ < 0.35; very thin grey lines); intermediate links (0.35 < C_i,j_ < 0.50; thin green lines); strong links (0.50 < C_i,j_ < 0.65; dark blue thick lines) and very strong links (C_i,j_ > 0.65; magenta very thick lines). To represent how network organization changes with fatigue across exercise segments (Figures 3, 4) we use a different links strength classification: weak links (0.45 < C_i,j_ < 0.55; very thin grey lines), intermediate links (0.55 < C_i,j_ < 0.65; thin green lines), strong links (0.65 < C_i,j_ < 0.75; dark blue thick lines) and very strong links (C_i,j_ > 0.75; magenta very thick lines). Links corresponding to cross-correlation values C_ij_ < 0.2 (Figure 2) and C_ij_ < 0.45 (Figures 3, 4) are not shown in the network maps as they are close to the significance threshold. Note that the significance threshold at 95% confidence level for network links strength during Rest is Th_rest_ = 0.12 and during Exercise is Th_exercise_ = 0.116.

To visualize the hierarchical organization of inter-muscular network interactions among muscle fibers (frequency band F_i_) from different muscles, the entire multiplex networks for Rest, Exercise 1, Exercise 2 and Exercise 3 are presented separately as sub-network maps for all pairs of muscles and distinct modules within the sub-networks. Each sub-network map corresponds to a muscle pair for a given protocol segment and follows the same color-code as in the original network. We obtain two types of sub-networks: 1) same-type muscle sub-networks (LegL-LegR and BackL-BackR; Figures 2, 3), and 2) sub-networks of different muscle types (LegL-BackL, LegL-BackR, LegR-BackL and LegR-BackR; Figure 4). For all sub-networks, we show selected network modules for frequency bands F2, F3, F5, F8 and F10 that represent the activation of type I, type IIa and type IIb muscle fibers. We quantify the hierarchical organization and stratification of links strength within the distinct modules in order to 1) understand how distinct muscle fibers from different muscles coordinate their activation and cross-communicate as a network to support posture during Rest and facilitate movement during Exercise, and to 2) associate network structure with the specific role (major, supportive or compensatory) different muscle pairs play during exercise and with accumulation of fatigue.

To further dissect inter-muscular interactions, we obtain stratification profiles of links strength for each sub-network, and we track how the stratification profiles are modulated with fatigue during repeated exercise bouts. For each protocol bout and all sub-network representing different pairs of muscles, we calculate the average links strength for distinct modules within the sub-network (corresponding group-averaged cross-correlation matrix in Figure 1D). Considering that each sub-network represents the interactions between EMG frequency bands for a given pair of muscles, we analyze the following network modules of cross-frequency coupling in each sub-network: *Low-low* frequencies [(F1-F2)—(F1-F2)] bands interactions: average value of the coupling (matrix elements in Figure 1D) between frequency bands (F1-F2) from one muscle with (F1-F2) from another muscle; *Low-intermediate* frequencies [(F1-F2)—(F3 … F7)] bands interactions: average value of the coupling between frequency bands (F1-F2) from one muscle with (F3, F4, F5, F6, F7) from another muscle; *Low-high frequencies* [(F1-F2)—(F8 … F10)] bands interactions: average value of the coupling between frequency bands (F1-F2) from one muscle with (F8, F9, F10) from another muscle; *Intermediate-intermediate* frequencies [(F3 … F7)—(F3 … F7)] bands interactions: average value of the coupling between frequency bands (F3, F4, F5, F6, F7) from one muscle with (F3, F4, F5, F6, F7) from another muscle; Intermediate-high frequencies [(F3 … F7)—(F8 … 10)] bands interactions: average value of the coupling between frequency bands (F3, F4, F5, F6, F7) from one muscle with (F8, F9, F10) from another muscle; *High-high* frequencies [(F8 … F10)—(F8 … F10)] bands interactions: average value of the coupling between frequency bands (F8, F9, F10) from one muscle with (F8, F9, F10) from another muscle (Figures 2C, 3C, 4B).

**FIGURE 7:**
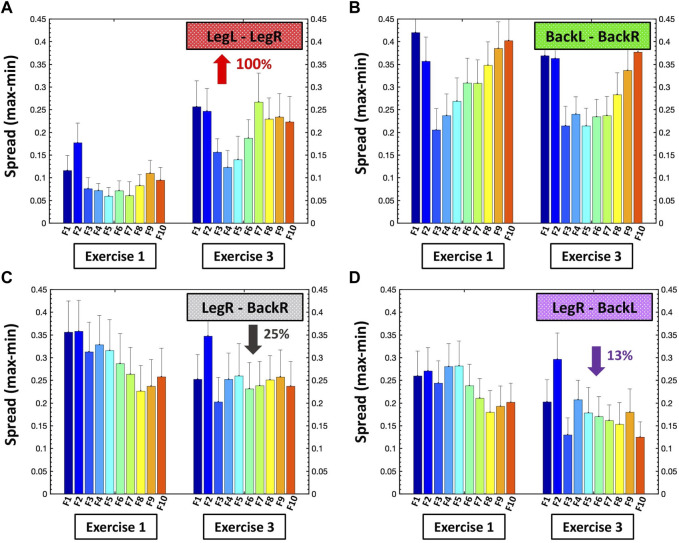
Degree of stratification in the links strength profiles for all modules in the same-type and different-type muscle sub-networks. Bar plots quantify the spread of links strength profiles for all ten modules in the **(A)** LegL-LegR, **(B)** BackL-BackR, **(C)** LegR-BackR and **(D)** LegR-BackL sub-networks (shown in Figures 5A,B, 6A,B) during Exercise 1 and Exercise 3. Each bar represents the spread of the ten links in each sub-network module, measured as the difference between the strongest and weakest link (Section 2.8, Methods) — e.g., the dark blue bar corresponding to F1 for Exercise 1 in **(A)** shows the links spread in the profile of the first module (group of ten links) in the LegL-LegR sub-network during Exercise 1 (Figure 5A, right panel). Distinct evolution is observed for the profiles spread of the modules within the four major sub-networks with repeated exercise bouts: **(A)** the links spread of the profiles for all ten modules in the LegL-LegR sub-network is initially low during Exercise 1 (homogenous profiles) and increases ≈100% during Exercise 3, which is in contrast to the evolution of the profiles in the second same-type muscle BackL-BackR sub-network shown in **(B)** where links spread is already high during Exercise 1 and does not increase with accumulation of fatigue during Exercise 3, reflecting different muscle fiber composition of the Back muscles and a different supportive (postural) role of the sub-network formed by the Back muscles in the squat movement. For the different-type muscle sub-networks LegR-BackR and LegR-BackL in **(C,D)** the profile spread for all ten sub-network modules is high during Exercise 1 and decreases during Exercise 3—a behavior different than the one observed in **(A)** and **(B)**, and related to the compensatory role of these two sub-networks in the squat movement with accumulation of fatigue.

Finally, we quantify the heterogeneity of each sub-network as the degree of stratification (spread) of the profile for the average links strength for all sub-network modules, and how the stratification profiles of all sub-networks change with fatigue during exercise bouts, by taking the difference between the strongest and weakest link in each module of a given sub-network (i.e., largest and smallest cross-correlation value C for each row in the group-averaged cross-correlation matrix corresponding to a given pair of muscles, Figure 1B) (Figure 7).

### 2.9 Statistical tests

Statistical analyses were performed using SPSS (version 23; SPSS, Inc.). All data were tested for normality by using a Shapiro-Wilk test. To assess changes between protocol segments in coupling strength for distinct network modules (Figures 2C, 3C, 4B, 5, 6) as well as in sub-networks heterogeneity (Figure 7), we perform a Wilcoxon matched-pairs test. To investigate changes in links strength within modules (Figures 5, 6) we use the Friedman ANOVA test. We establish an alpha-level of 0.05 for all statistical tests.

## 3 Results

We identify and characterize inter-muscular network of muscle fibers interactions during Rest and Exercise, and investigate how synchronous activity of muscle fibers and network organization changes across repeated exercise segments with accumulation of fatigue. We consider myoelectrical activity from muscles with different function—left and right leg vastus lateralis muscles (Leg-Left and Leg-Right), and left and right lower back erector spinae muscles (Back-Left and Back-Right). EMG signals from the four muscles were simultaneously recorded during supine rest and squat test performed till exhaustion (Section 2.2, Methods; Figures 1A–C). To identify how distinct muscle fibers dynamically coordinate and synchronize their activation, and integrate as a network across muscle groups to facilitate resting state or movement, we decompose the recorded EMG signals in ten frequency bands corresponding to the discharge of different motor neurons and related to them muscle fibers (Section 2.4, Methods; (Grimby et al., 1979, 1981; Wakeling et al., 2001; Dreibati et al., 2010; Rosenblum et al., 2021). Thus, in our network each muscle is characterized by ten network nodes (Figure 1D), which interact with the nodes representing fiber dynamics in the other muscles leading to complex network organization (Figure 2A). We then quantify pair-wise coupling between the spectral power of frequency bands, and derive network interactions among distinct muscle fiber types across all muscles. Group-averaged cross-correlation matrices representing intermuscular muscle fiber interactions for Rest and Exercise segments show complex heterogeneous structure (Figure 1D), where matrix elements represent the pairwise coupling strength between the ten frequency bands for each pair of muscles (sub-networks LegL-LegR, LegL-BackL, LegL-BackR, LegR-BackL, LegR-BackR and BackL-BackR) during all protocol segments (Rest, Exercise 1, Exercise 2 and Exercise 3).

Our results demonstrate that each state (rest or squat exercise) is uniquely characterized by a specific ensemble of interaction sub-networks (distinct heterogenous matrices) representing all pairs of muscle groups, where each sub-network exhibits a unique pattern of synchronization (hierarchical structure in network links strength) among myoelectrical rhythms representing muscle fibers with different physiological functionality within each muscle group. Specifically, intermediate and high frequency myoelectrical rhythms are strongly coupled for the BackL-BackR interaction sub-network during supine Rest (matrix elements in warm colors, Figure 1D), while all frequency rhythms (all type muscle fibers) are decoupled for the LegL-LegR and Leg-Back sub-networks (matrix elements in dark blue colors below the physiologically significant threshold, Figure 1D and Section 2.6, Methods). In contrast, during Exercise there is significantly elevated degree of coupling and synchronization in the activity of muscle fibers for all pairs of muscle groups (coupling strength above the threshold of physiological significance; matrix elements in warm colors, Figure 1D), with distinct matrix structure for the LegL-LegR, BackL-BackR and Leg-Back interaction sub-networks. During supine rest the average coupling strength in all inter-muscular sub-networks is below the significance threshold except for the BackL-BackR sub-network (Figure 1E), reflecting higher muscle tone in Back muscles necessary to maintain supine rest associated with higher fraction of type I slow (low frequency) muscle fibers. Note that the average coupling strength for all sub-networks (pairs of muscle groups) during Exercise 1 increases dramatically multiple times, with significantly higher coupling strength for the same-type muscle sub-networks LegL-LegR and BackL-BackR (Figure 1E). For repeated bouts of Exercise 1, 2 and 3 we find a significant reorganization in all inter-muscular sub-networks (pairs of muscles), indicating changes in the coordination and degree of synchronous activation among muscle fibers across muscles with accumulation of fatigue.

These observations indicate that distinct physiological states such as rest, exercise and fatigue there are characterized by a unique signature of network communication underlying the coordination of muscle fiber activity across muscles. With transition from rest to exercise, inter-muscular interactions exhibit: 1) a dramatic increase in the coupling strength for all sub-networks, with stronger interactions among muscle fibers within same-type muscle sub-networks (LegL-LegR and BackL-BackR) compared to sub-networks of different muscle type (LegL-BackL, LegL-BackR, LegR-BackL and LegR-BackR) (Figure 1E), and 2) specific signatures of cross-frequency communication for each sub-network in order to synchronize activation among distinct muscle fibers and coordinate force generation across muscles with different functions. For repeated exercise segments, our analyses demonstrate a complex hierarchical reorganization in the coupling strength of muscle fibers where distinct sub-networks within the entire inter-muscular network respond in a targeted way to overcome reduced motor neuron excitability with accumulation of fatigue—e.g., while the coupling strength of same-type muscle sub-networks is reduced due to fatigue, sub-networks of different-type muscles increase their strength of interactions (Figure 1D). These general observations reflect the existence of a previously unrecognized mechanism of inter-muscular interactions mediated through synchronous activation of distinct muscle fiber types.

### 3.1 Inter-muscular interaction networks: Rest vs. exercise

To quantify how inter-muscular networks of muscle fiber interactions change with transition from rest to exercise, we map the cross-correlation matrices in Figure 1D intro dynamic networks where nodes correspond to distinct myoelectrical rhythms associated with the activity of distinct muscle fibers, and links represent the coupling strength among rhythms (frequency bands F_i_) across muscles (Figure 2A). During supine rest the inter-muscular network is characterized by physiologically significant synchronization of muscle fibers only within the BackL-BackR sub-network, and absence of coupling among muscle fibers in the LegL-LegR and Leg-Back sub-networks. This reflects higher muscle tone and synchronous muscle fiber activation in the back compared to the leg muscles due to the distinct histochemical characteristics and postural role of the back muscles under resting conditions. With transition to Exercise a complex topology of sub-networks representing pairs of same- and different-type muscle groups emerges, where the same-type muscle LegL-LegR and BackL-BackR sub-networks exhibit strong and intermediate links, in contrast to the sub-networks representing pairs of different-type Leg-Back muscles with weaker coupling among muscle fibers (Figure 2A).

To probe the hierarchical structure of network links strength and how inter-muscular network organization changes with transition from rest to exercise, we dissect the entire network into separate sub-network modules for low (F1, F2, F3), intermediate (F4, F5, F6, F7) and high (F8, F9, F10) frequency EMG rhythms. We find that within same-type muscle LegL-LegR and BackL-BackR sub-networks, all network modules show consistent stratification in links strength—i.e., links are stronger for matching frequency bands and gradually decline for more distant frequency bands from the two muscle groups in each sub-network. This effect is more pronounced for the BackL-BackR sub-network during both rest and exercise, where the spectral power dynamics of low-frequency (F2-F3) muscle fibers in the BackL are highly synchronized with low frequency fibers in BackR; intermediate frequencies (F5) in BackL muscle communicate to intermediate and high frequencies in BackR, and high frequencies (F8-F10) in BackL communicate to intermediate and high frequencies in BackR, representing the strongest couplings within the BackL-BackR sub-network (Figure 2B).

To further dissect inter-muscular muscle fiber interactions and quantify the stratification patterns observed for the links strength of the modules embedded in LegL-LegR and BackL-BackR sub-networks (Figure 2B), we calculate the average coupling strength for distinct sub-groups of links within modules (bar charts in Figure 2C). We find that a characteristic profile of network links for the modules in LegL-LegR and BackL-BackR sub-networks. During rest, the BackL-BackR sub-network exhibits a stratification profile with significant decline in average links strength for the sub-network modules corresponding to low-low [(F1-F2)—(F1-F2)], low-intermediate [(F1-F2)—(F3, … ,F7)] and low-high [(F1-F2)—(F8, … ,F10)] frequency bands interactions (first three bars of the stratification profile in Figure 2C), and significantly stronger links (higher bars) for the sub-network modules corresponding to intermediate-intermediate [(F3, … ,F7)—(F3, … ,F7)], intermediate-high ([(F3, … ,F7)—(F8, … ,F10)] and high-high [(F8, … ,F10)—(F8, … ,F10)] frequency bands interactions (last three bars of the BackL-BackR stratification profile in Figure 2C). During Exercise, we find a significant increase in coupling strength for LegL-LegR (≈700%; Wilcoxon *p* values <0.001) and BackL-BackR (≈60%; Wilcoxon *p* values <0.05), and a similar stratification profile in links strength for the different sub-network modules in both LegL-LegR and BackL-BackR sub-networks, that is more pronounced for the BackL-BackR sub-network. Note that during exercise the stratification in the profile of the LegL-LegR sub-network is reduced compared to the BackL-BackR sub-network (Figure 2C) due to significant targeted increase in coupling strength for low-intermediate [(F1-F2)—(F3, … ,F7)] (Wilcoxon *p* = 0.04) and low-high [(F1-F2)—(F8, … ,F10)] (Wilcoxon *p* = 0.03) frequency bands interactions.

### 3.2 Inter-muscular interaction networks: Reorganization with fatigue

Further, we ask the question of how distinct muscle fibers within different muscle groups dynamically coordinate and synchronize their activation across exercise segments with accumulation of fatigue. Specifically, we investigate how network organization and the profiles of network links strength within sub-networks and sub-network modules observed for Exercise 1 (Figure 2) change with repeated exercise bouts (Exercise 2 and 3) for sub-networks of same-type (Figure 3) and different-type muscles (Figure 4).

Our analyses show that with repeated squat exercise bouts, the global inter-muscular network undergoes complex hierarchical reorganization where sub-networks representing pairs of muscles with different function exhibit differentiated response to fatigue (Figure 3A). Specifically, sub-networks of same-type muscle interactions (LegL-LegR and BackL-BackR), which serve as main facilitators of squat movement, are characterized by reduced connectivity and significant decline in network links strength. In contrast, sub-networks of different muscle-types (four Leg-Back sub-networks), which play compensatory role in squat movement, exhibit increased connectivity and stronger links of interaction among muscle fibers. These findings indicate significant change in the way muscle fiber synchronize their activation across major muscles with different functions and integrate as a network to adjust muscle function to fatigue.

Same-type muscle sub-networks: To better understand how inter-muscular networks of muscle fiber interactions reorganize with fatigue, we next study the hierarchical organization of links strength in individual sub-network modules. We find that all modules in the same-type muscle LegL-LegR sub-network undergo remarkable changes due to pronounced fatigue effects provoked by Exercise 1 (squats movements performed to exhaustion). During Exercise 2, links in the LegL-LegR sub-network modules are characterized by 1) significantly reduced coupling strength among frequency bands and 2) increased stratification in links strength where links are stronger for matching frequency bands and gradually decline for more distant frequency bands from the two muscle groups in the sub-network. Specifically, the stratification in links strength increases for all modules in the LegL-LegR sub-network during Exercise 2 and 3 —from very strong and strong links during Exercise 1 to the entire spectrum of very strong to intermediate and weak links during Exercise 2 and 3). Notably, no significant changes are observed in the links strength organization of the LegL-LegR sub-network during Exercise 3, indicating that the sub-network reached the minimum level of inter-muscular interactions during Exercise 2 (Figure 3B). In contrast, we find that the same-type muscle BackL-BackR sub-network remains unchanged during Exercise 2, with same coupling strength and level of stratification of links in all modules as in Exercise 1, indicating a different response to fatigue in the way different muscle fibers synchronize activity in the LegL-LegR and BackL-BackR sub-networks. Further, while BackL-BackR sub-network interactions become weaker during Exercise 3, the link strength stratification for all modules remains the same—i.e., coupling strength is homogeneously reduced for all BackL-BackR sub-network links in contrast to the LegL-legR sub-network.

To quantify the observed hierarchical organization of links strength in network modules embedded in the same-type muscle LegL-LegR and BackL-BackR sub-networks, we next obtain the link strength stratification profiles corresponding to each sub-network, and we track the evolution of the stratification profiles for consecutive exercise bouts in response to accumulation of fatigue (Figure 3C). Our empirical observation show that during Exercise 1 both LegL-LegR and BackL-BackR sub-networks exhibit similar structure for their stratification profiles—i.e., decline in average links strength for the sub-network modules corresponding to low-low [(F1-F2)—(F1-F2)], low-intermediate [(F1-F2)—(F3, … ,F7)] and low-high [(F1-F2)—(F8, … ,F10)] frequency bands interactions (first three bars in each stratification profile, Figure 3C), and much stronger links (higher bars) for the sub-network modules corresponding to intermediate-intermediate [(F3, … ,F7)—(F3, … ,F7)], intermediate-high [(F3, … ,F7)—( F8, … ,F10)] and high-high [(F8, … ,F10)—(F8, … ,F10)] frequency bands interactions (last three bars in each stratification profile, Figure 3C). Notably, the stratification in links strength between the different modules in the BackL-BackR during Exercise 1 is significantly more pronounced compared to the LegL-LegR sub-network—i.e., bigger differences between the bars in the interaction profile of the BackL-BackR sub-network.

We find that with accumulation of fatigue during Exercise 2 and Exercise 3, the average links strength significantly declines for all modules in the LegL-LegR sub-network which plays the major role in facilitating squat movements. Further, this decline in links strength is associated with increased stratification in the interaction profile of the LegL-LegR sub-network, reflecting higher dispersity in the average links strength of the modules embedded in the sub-network and more pronounced hierarchy in network organization (Figure 3C, left panel). Remarkably, with transition from Exercise 2 to Exercise 3 there is no further decline in the average links strength for all modules in the LegL-LegR sub-network, and the stratification profile remains the same. This indicates that already during Exercise 2 the LegL-LegR sub-network has reached the lowest possible physiological level of network coordination and degree of coupling among muscle fibers from the two leg muscles, and thus, the sub-network does not respond to further accumulation of fatigue during Exercise 3. These findings raise the question whether other sub-networks involving different pairs of muscles take over and compensate the lack of response in the LegL-LegR sub-network at very high levels of fatigue (see Figures 4, 6).

In contrast to the LegL-LegR sub-network, links strength in the modules of the BackL-BackR sub-network remain unchanged during Exercise 2, thus, preserving the same stratification profiles as in Exercise 1 despite the accumulation of fatigue. Our findings that with accumulation of fatigue during Exercise 2 interactions are reduced in the LegL-LegR sub-network which takes the major effort, while links strength and the stratification profile of the BackL-BackR sub-network remain unchanged, indicate the secondary and supportive role back muscles and their interactions play during squat movements leading to less exhaustion and steady network dynamics. Further, compared to leg muscles, back muscles have different muscle fiber composition (higher ratio of slow type I muscle fibers) and histochemical characteristics (higher amounts of glycogen deposits and larger amounts of mitochondria involved in back muscle energy conversion) (Cagnie et al., 2015; Deshmukh et al., 2021), that relate to the different reaction time and higher resistance to fatigue during Exercise 2 as demonstrated by the BackL-BackR sub-network dynamics. Remarkably, links strength in the modules of the BackL-BackR sub-network is reduced significantly only during Exercise 3, however, preserving the form of the stratification profile—an effect of homogeneous reduction in links strength across all modules that is specific for this sub-network (Figure 3C, right panel). Our observations indicate markedly different response to fatigue of same-type muscle LegL-LegR and BackL-BackR sub-networks, where network reorganization and the associated stratification profile of links strength undergo very different trajectories corresponding to 1) the different muscle fiber composition of the Leg and Back muscles, 2) the very different role these muscles play during squat movement, and 3) the way muscle fibers within these muscle groups coordinate their activity and interact in response to accumulation of fatigue.

Sub-networks of different muscle types: Since our observation in Figure 3 demonstrate that the LegL-LegR and BackL-BackR sub-networks exhibit different characteristics (connectivity and links strength stratification) and response to fatigue that are associated with their specific role (major vs. supportive) during squat movements, we next study muscle fibers interactions between pairs of different-type muscles (Leg-Back) and quantify their contribution to the hierarchical structure of the global inter-muscular network. We find that during exercise each network module in the LegR-BackR and LegR-BackL sub-networks is characterized by different average link strength and by a specific topological organization for links of different strength (Figure 4A) — stronger and more heterogenous links in the F2 module compared to weaker and more homogeneous links in the F10 module during Exercise 1, indicating even more complex pattern of cross-communication and synchronization of muscle fiber activity in different-type muscle sub-networks compared to the same-type muscle sub-networks (Figure 3). With transition to consecutive exercise bouts Exercise 2 and Exercise 3, the average link strength increases, and the topological organization of links with different strength in each network module changes, indicating a hierarchical reorganization across modules in both LegR-BackR and LegR-BackL sub-networks in response to accumulation of fatigue that is different compared to the response observed for interactions in the same-type muscle sub-networks.

To quantify the response of muscle fiber interactions within the different-type muscle sub-networks to fatigue, we obtain stratification profiles representing the average links strength for all network modules embedded in the LegR-BackR and LegR-BackL sub-networks, and we track the evolution of these profiles with repeated exercise bouts (Figure 4B). We find that both LegR-BackR and LegR-BackL sub-networks exhibit similar interaction profiles during Exercise 1 — i.e., significant decline in average links strength for the sub-network modules corresponding to low-low [(F1-F2)—(F1-F2)], low-intermediate [(F1-F2)—(F3, … ,F7)] and low-high [(F1-F2)—(F8, … ,F10)] frequency bands interactions (first three bars in the profile follow an up-down-down pattern, Figure 4B), and weaker links for the sub-network modules corresponding to intermediate-intermediate [(F3, … ,F7)—(F3, … ,F7)], intermediate-high [(F3, … ,F7)—( F8, … ,F10)] and high-high [(F8, … ,F10)—(F8, … ,F10)] frequency bands interactions that follow the same up-down-down pattern (last three bars in the profile, Figure 4B). With accumulation of fatigue for repeated squat bouts Exercise 2 and Exercise 3 the general shape of the interaction profiles for both LegR-BackR and LegR-BackL sub-networks is preserved, however, the average links strength corresponding to the sub-network modules of intermediate-intermediate, intermediate-high and high-high frequency bands (last three bars in the profiles, Figure 4B) significantly increases (comparing Exercise 2 vs. Exercise 1; Wilcoxon test *p*-value *p* < 0.04), leading to reduced stratification in coupling among muscle fibers. Remarkably, the stratification profiles for the different-type muscles sub-networks in Figure 4B and their modulation with fatigue are markedly different compared to the interaction profiles of the same-type muscles sub-networks shown in Figure 3C—while the average links strength in the modules of same-type muscle sub-networks decline with fatigue, link strength increases in the different-type muscle sub-networks. This reflects the compensatory role different-type muscles sub-networks play in the mechanism of maintaining leg-trunk coordination with accumulation of fatigue, in contrast to the same-type muscle sub-networks which absorb the main effort during squat movement. We find a similar network structure and reorganization across exercise bouts is observed for the other two sub-networks LegL-BackL and LegL-BackR of different muscle types (not shown in Figure 4) — a consistency that demonstrates the presence of a basic physiologic law regulating muscle fiber interactions across muscles with different functions.

### 3.3 Patterns in inter-muscular network links strength: Reorganization with fatigue

The results of our analyses shown in Figures 2–4 reveal complex dynamic patterns of muscle fiber synchronous activity in rest and exercise, and a structured hierarchical organization of distinct network modules and sub-networks through which muscle fiber interactions integrate into a global inter-muscular network to facilitate coordination and synergy among muscles with different composition and function during movement. The stratification profiles of average links strength within separate sub-networks and network modules, however, present a course-grained level of network structure and dynamics with transition from rest to exercise and their evolution with accumulation of fatigue. To probe the fine structure within the hierarchical organization of the inter-muscular network of muscle fiber interactions, we analyze all individual links for all modules within each sub-network: two same-type sub-networks (LegL-LegR and BackL-BackR, Figure 5), and four different-type sub-networks (LegR-BackR, LegR-BackL, LegL-BackL and LegL-BackR, Figure 6). We find that links within individual modules (10 links per module representing the degree of coupling between a frequency band F_i_ from one muscle with all frequency bands of the other muscle in a given sub-network, Methods) exhibit unique profiles specific for each sub-network (10 modules and 100 links per sub-network, Methods), thus, representing a marker of the function distinct sub-networks play in coordinating interactions among all muscles in the global network.

Same-type muscle sub-networks: Our analyses of the same-type muscle LegL-LegR sub-network show complex dynamics of the interactions among muscle fibers (represented by frequency bands F_i_) characterized by unique profile of links strength for each sub-network module (Figure 5A, right panels). Specifically, during Exercise 1 a profile with declining links strength characterizes the sub-network module of interaction between F1 frequency band of LegL with all ten frequency bands of LegR, as shown by the first group of ten bars in Figure 5A (top right panel). This contrasts with modules F9 and F10 characterized by increasing links strength, while modules F3 and F4 exhibit almost flat profiles during Exercise 1. Further, during Exercise 2 and Exercise 3 the strength of all links significantly declines across all ten modules in the LegL-LegR sub-network, while the stratification in the links strength profile of each sub-network module significantly increases (100% increase for Exercise 3 vs. Exercise 1, Figure 7A) in response to accumulation of fatigue with repeated exercise bouts.

Notably, modules in the BackL-BackR sub-network exhibit a similar shape for their links strength profiles as modules in the LegL-LegR sub-network, however, with significantly higher stratification of links strength within each module as well as less pronounced decline in links strength with repeated exercise (decline in links strength is observed only for some modules during Exercise 3, Figure 5B right panels). Remarkably, in contrast to the LegL-LegR sub-network, the degree of links strength stratification of the profile for each module within the BackL-BackR sub-network does not significantly change with repeated exercise bouts (Figure 7B). These empirical findings demonstrate that same-type muscle sub-networks, which are the major contributors to squat movement, exhibit similar hierarchical organization (profiles of links strength) within sub-network modules, while the links strength stratification within each module and the degree of decline of links strength in response to accumulation of fatigue is markedly different. This reflects physiological differences related to muscle fiber composition of leg and back muscles, and essentially different role of the two sub-networks during squat movements—i.e., major dynamic movement generation role of the LegL-LegR sub-network vs. secondary supportive stabilization role of the BackL-BackR sub-network.

Sub-networks of different muscle type: We next extend our analyses of the hierarchical organization of the inter-muscular network at the level of individual links representing muscle fibers interactions for all modules in the different-type muscle sub-networks (LegR-BackR, LegR-BackL, LegL-BackL and LegL-BackR). Considering the LegR-BackR sub-network (muscles on the same side of the body), we find that the topology of links strength changes with repeated exercise bouts (Figure 6A, left panels). Specifically, during Exercise 1 the modules in the LegR-BackR sub-network form a complex hierarchy of network links strength where links within each module exhibit a similar profile with declining strength—i.e., the degree of coupling for all frequency bands in the LegR muscle declines for increasing frequency bands in the BackR muscle (Figure 6A, top right panel). With repeated squat bouts Exercise 2 and Exercise 3, the shape of the links strength profiles for all modules in the LegR-BackR sub-network gradually changes in response to accumulation of fatigue, where the interactions of all frequency bands in the LegR muscle with the F1 and F2 bands of the BackR muscle (first two bars in each module of ten bars) significantly decline (dashed dark blue guiding line, Figure 6A right panels), while the strength of interactions with the intermediate and high frequency bands in the BackR muscle significantly increases (dashed light blue and red guiding lines, Figure 6A right panels). Notably, the average link strength in each module of the sub-network increases for Exercise 2, and remains unchanged for Exercise 3, which contrasts with the response observed for the modules in the same-type muscle LegR-LegL and BackL-BackR sub-networks in Figure 5.

Further, with find that modules in the muscles LegR-BackL sub-network (muscles on the opposite side of the body) exhibit 1) similar shape for their links strength profiles as the modules in the LegR-BackR sub-network; 2) similar increase of the average link strength during Exercise 2; and 3) similar modulation of the profiles shape during Exercise 2 and 3 in response to fatigue (Figure 6B, right panels). The stratification of links strength profiles in the modules of both different-type muscle sub-networks LegR-BackR and LegR-BackL gradually declines with accumulation of fatigue during Exercise 2 and Exercise 3. In contrast to the response to fatigue of the same-type muscle sub-networks LegL-LegR and BackL-BackR, where the average links strength declines and the shape of the links strength profile for all modules is preserved (Figure 5), we observe a significant increase in the average links strength and modulation of the profiles (i.e., decline in profiles stratification) for all modules in the different-type muscle sub-networks LegR-BackR and LegR-BackL during Exercise 2 and 3 (Figures 7C,D). These findings indicate a very different compensatory role of the LegR-BackR and LegR-BackL sub-networks compared to the LegL-LegR and BackL-BackR sub-networks, to decelerate neuromuscular failure and maintain muscle coordination. Remarkably, we find that a similar network structure and reorganization of links strength profiles across exercise bouts is observed for the other two sub-networks LegL-BackL and LegL-BackR of different muscle types (not shown in Figure 6)—consistency that confirms the physiologic significance of the results and the relation of the derived network of muscle fiber interactions to endogenous mechanisms of regulation.

## 4 Discussion

The present study investigates inter-muscular interactions among rhythms of myoelectrical activation corresponding to the activity of different type muscle fibers across muscles with different muscle fiber composition and distinct functions. We address the fundamental question of how different type muscle fibers dynamically synchronize and integrate as a network across muscles to facilitate coordinated movements, and how inter-muscular network interactions reorganize with transition from rest to exercise and respond to accumulation of fatigue during consecutive exercise bouts of squat movements.

Uncovering the nature of interactions of among muscle fibers is key to understanding the mechanisms regulating the function of individual muscles and why/how muscle groups coordinate to facilitate variety of movements. One possible hypothesis is that all muscle fibers in a given muscle activate simultaneously to produce a global response to external inputs. An alternative hypothesis is that muscle fibers of different types synchronize activity with linear delays corresponding to their histochemical and mechanical characteristics—i.e., synchronous activation occurring first between slow muscle fibers, followed with some time-delay by synchronization between intermediate and fast muscle fibers. A third hypothesis that at every state and type of movement, muscle fibers of all types simultaneously cross-communicate with each other within a muscle as well as across muscle groups—a highly complex behavior with rich patterns of interaction and network integration of muscle fibers activity that allows for fine-tuned coordination for a variety of movements. Our empirical results confirm this most complex scenario.

Given that all muscle fiber types simultaneously interact through different degrees of coupling during movement, one possible outcome could be that each movement involving a specific group of muscles is represented by unique network of muscle fiber interactions, and thus, an infinite number of distinct networks would be needed to characterize/represent the gigantic phase-space of different human movements and behaviors. Alternatively, while different movements may involve diverse muscles, the underlying network of muscle fiber interactions may be characterized by general principles of hierarchical organization (sub-networks and modules) that do not depend on the diversity of the muscles involved, but rather represent the role of the muscles during the movement (major, secondary or compensatory). Thus, an infinite variety of movements involving various combinations of muscles could be represented by a few classes of muscle fiber interaction networks with specific hierarchical organization. Our investigations demonstrate that the uncovered hierarchical organization of muscle network interactions and their reorganization with transition from rest to exercise and fatigue, do not depend on the specific movement but rather reflect the major, secondary or compensatory role pairs of muscles with different characteristics/function play in facilitating a given movement.

We find first empirical evidence that muscle fibers of different types synchronize their activity across muscle groups following distinct dynamic patterns of cross-frequency communication that depend on muscle characteristics and on the role of muscles in a given movement. We discover that the structure and hierarchical organization of the inter-muscular interaction network are uniquely associated with distinct physiological states of rest, exercise and fatigue. Further, we discover that each pair of muscles in the global inter-muscular network forms a sub-network characterized by specific signatures of cross-frequency communication to synchronize activation among distinct muscle fiber types, and that each sub-network comprises multiple modules with unique profiles of links strength stratification that follow specific evolution paths in the process of adapting to accumulation of fatigue during repeated exercise. While earlier studies have focused on myoelectrical responses of individual muscles (e.g., change in EMG amplitude and dynamics; (Cardozo et al., 2011; Politti et al., 2016), the Network Physiology framework we utilize in this study demonstrates that the coupling strength (degree of synchronization) between myoelectrical rhythms and the topology of their network interactions change in response to transitions across physiological states, and adapt in a coordinated way among sub-networks and modules to increasing levels of fatigue. The reported here empirical results open the perspective for an entire new class of biomarkers based on network interactions among muscle fibers across muscle groups to characterize effects of different type exercises, implement optimal training programs, assess fitness status and effectiveness of muscle injuries treatment and rehabilitation.

We demonstrate the existence of preferred pathways of cross-frequency communication across leg and back muscles that uniquely characterize the inter-muscular interaction network for the distinct physiological states of supine rest and squat exercise. We observe that the global inter-muscular network involving interactions among all muscles drastically reorganizes with transition from rest to exercise (Figure 2A). In contrast to rest, the inter-muscular network during exercise exhibits high connectivity with stronger network links and a hierarchical organization of sub-networks with distinct characteristics to coordinate force generation under higher physiological demands (Figure 2A).

The network of inter-muscular cross-frequency coupling among myoelectrical rhythms associated with different muscle fiber types is sparse during rest, where all interaction sub-networks are absent except the BackL-BackR sub-network. Back muscles (left and right erector spinae) are mainly composed of slow type I fibers (Cagnie et al., 2015) and have postural role under supine resting conditions, while leg muscles (left and right vastus lateralis) have higher percentage of fast type II fibers (Vromans and Faghri, 2018; Nederveen et al., 2020), play major dynamic role during squat movements and remain not active during supine rest. Thus, back muscles exhibit higher muscle tone than leg muscles during supine rest. Our analyses show that, in addition to higher back muscle tone, rest is characterized by a high degree of synchronous activation among muscle fibers from the left and right back muscles and by a BackL-BackR sub-network structure of distinct interaction modules with different average links strength (Figure 2B) that follow a unique link strength stratification profile (Figure 2C). These findings of complex cross-frequency network communication among muscle fibers are a new hallmark of muscles coordination during rest that does not depend on the activation level of each separate muscle—note, that two systems with low level of activation could exhibit high degree of coordination, and thus, strong coupling (Lin et al., 2020; Rizzo et al., 2020, 2022).

Further, BackL-BackR sub-network interactions during rest exhibit unique hierarchical organization with a specific stratification profile formed by the average links strength of the distinct modules embedded in the sub-network, where the modules corresponding to low-low [(F1-F2)—(F1-F2)], intermediate-high [(F3-F7)-(F8-F10)] and high-high [(F8-F10)—(F8-F10)] frequency bands interactions exhibit dominant links strength (Figure 2C). Since the average conduction velocity of an active motor unit relates to the muscle fiber type (Casolo et al., 2022), and changes in EMG spectral properties are linked to changes in the average conduction velocity (Farina, 2008; Von Tscharner and Nigg, 2008), the uncovered links strength stratification profile in the BackL-BackR sub-network during rest, indicates that at this state slow type I muscle fibers in the BackL muscle predominantly communicate with the same type I fibers in the BackR muscle, while intermediate type IIa fibers communicate with both type IIa and fast type IIb fibers. A feasible explanation of the links strength stratification profile is that muscle fibers from different muscles synchronize better when they have similar histochemical characteristics, and thus, sub-network modules corresponding to low-low, intermediate-intermediate and high-high frequency bands exhibit stronger links. The observed high cross-frequency communication between type IIa and IIb muscle fibers (strong intermediate-high frequency links in the profile, Figure 2C) may also be related to their similar histochemical characteristics (Deshmukh et al., 2021).

Remarkably, a similar hierarchical organization of modules with different links strength and stratification profile are observed for the BackL-BackR sub-network during Exercise 1 (Figure 2C), however, with stronger global coupling strength and a more pronounced increase in the intermediate-intermediate frequency coupling. Similar to rest, the shape of the stratification profile of inter-muscular links strength for the BackL-BackR sub-network during exercise reflects stronger interactions for links between same frequency bands (Figure 2B), where each muscle fiber type in BackL communicates predominantly with the same type of muscle fiber in BackR. This basic law of muscle fiber cross-communication accounts for the high degree of stratification (spread) observed in the links strength profile of the BackL-BackR sub-network in both rest and exercise (Figure 7B).

In contrast to BackL-BackR network interactions, links strength in the LegL-LegR sub-network during rest is below the threshold of physiological significance (Figure 2C; black dotted line), reflecting the absence of significant inter-muscular interactions of muscle fibers between leg muscles. Notably, the characteristic stratification profile observed in the BackL-BackR sub-network during exercise is also present for the LegL-LegR sub-network, however, with higher coupling strength for low-intermediate and low-high frequency band interactions. This indicates that during exercise, unlike the BackL-BackR sub-network, slow type I muscle fibers in the LegL muscle can communicate (synchronize activation) with intermediate type IIa and fast IIb muscle fibers in the LegR muscle, leading to a links strength profile with reduced stratification (spread) (Figure 7A). The reported differences between the BackL-BackR and LegL-LegR sub-networks reflect the distinct histochemical characteristics and specific role these sub-networks play in squat movements—while vastus lateralis leg muscles have primary motor role during squat movements, erector spinae back muscle have a secondary supportive role (trunk stability) (Khaiyat and Norris, 2018).

Importantly, we further demonstrate that inter-muscular networks of muscle fiber interactions undergo a particular reorganization path with repeated exercise bouts to overcome and compensate effects of accumulated fatigue. While same-type muscle sub-networks (LegL-LegR and BackL-BackR) become sparser with weaker links for repeated exercise (Figures 3, 5), sub-networks of different-type muscles (LegL-BackL, LegL-BackR, LegR-BackL and LegR-BackR) exhibit higher connectivity and stronger links (Figures 4, 6).

Notably, the links strength stratification profile for the modules in the LegL-LegR sub-network during Exercise 1 is consistently observed also for Exercise 2 and Exercise 3, however, with 1) significantly reduced coupling strength and 2) increased degree of stratification (spread; Figure 7A) due to a remarkable reduction in average links strength for the sub-network modules representing coupling between low-intermediate and low-high frequency bands (Figure 3C). These observations demonstrate that fatigue provokes reduction in the cross-frequency communication between different type muscle fibers in the LegL-LegR sub-network, while strongest interactions are mediated by same type muscle fibers.

Similarly to the LegL-LegR sub-network, the general shape of the links strength profile in the BackL-BackR sub-network observed for Exercise 1 does not change during Exercise 2 and Exercise 3, and both sub-networks exhibit reduction in links strength with fatigue (Figure 3C). However, for the BackL-BackR sub-network we find 1) delayed fatigue onset and less pronounced reduction in link strength for repeated exercise bouts compared to the LegL-LegR sub-network, and 2) no increase in stratification (spread) of the links strength profile due to a homogenous decrease of links strength for all modules within the sub-network (Figure 5B; Figure 7B). The delayed onset of fatigue on BackL-BackR interactions (i.e., only during Exercise 3) reflects the secondary/supportive role back muscles play during squat movements, as well as its fatigue-resistant histochemical characteristics (Cagnie et al., 2015) compared to leg muscles. As recently demonstrated, high initial levels of common input and high coordination between muscles (as observed here in the LegL-LegR and BackL-BackR sub-networks during Exercise 1) represent a neural constrain making it less likely to redistribute the neural drive across these muscles during fatiguing tasks (Rossato et al., 2022), and thus, to adapt to fatigue in ways other than to reduce coupling strength. This could explain the lack of ability of the same-type muscle LegL-LegR and BackL-BackR sub-networks to find more flexible coordination strategies with accumulation of fatigue but to reduce links strength across all modules in the sub-network.

Considering the different-type muscle LegR-BackR and LegR-BackL sub-networks, a different stratification profile of network links strength emerges for the modules in these sub-networks with remarkably weaker coupling strength during Exercise 1 (Figure 1E, Figure 4B), compared to the same-type muscle sub-networks (Figure 3C). LegR-BackR and LegR-BackL inter-muscular communication is predominantly mediated by low-low and intermediate-intermediate frequency band interactions. This indicates that slow type I and intermediate type IIa fibers in the leg muscle synchronize activity mainly with the corresponding type I and IIa fibers in the back muscle. Opposite to same-type LegL-LegR and BackL-BackR sub-networks where interactions decline with fatigue (Figure 3C), links strength profiles of the LegR-BackR and LegR-BackL sub-networks during Exercise 2 and 3 are characterized by 1) increase in average link strength with fatigue accumulation, and 2) decline in the stratification (spread) of the interaction profiles due to a concentrated increase of links strength for the modules representing intermediate-intermediate, intermediate-high and high-high frequency bands interactions (Figure 4B, Figures 7C,D). Such higher flexibility of the different-type muscle sub-networks in response to fatigue (i.e., ability to increase coupling strength with progression of fatigue) can be related to the fact that low initial common synaptic input between muscles allows for more flexible coordination strategies during fatiguing tasks (Rossato et al., 2022).

Our findings of complex hierarchically-structured network interactions of muscle fibers across topologically distant leg and back muscles align with recent works investigating motor neuron recruitment during exercise and inter-muscular coherence (Hug et al., 2021), which demonstrated that motor neurons from distant muscles (e.g., hamstrings and gastrocnemii) may receive common neuronal input during dynamic pedaling. Other works have reported the presence of common neural inputs also to other pairs of distinct muscles such as hamstrings and plantar flexors (Hug et al., 2011), as well as glutei and quadriceps during gait (Dominici et al., 2011). This suggests a possible mechanism where a fraction of motor neurons within a pool that innervates a given muscle interact with a fraction of the motor neurons that innervate another muscle, thus, forming a distinct functional cluster associated with a given movement, and that multiple functional clusters of motor neurons with differentiated role across distant muscles facilitate a broad range of movements. In other words, each motor unit may generate a force to assist movement in particular direction and, thus, the recruitment of specific groups of motor neurons across several muscles may be a strategy to comply with the mechanical demands of a given movement task (Hug et al., 2021). This may explain why different muscle fiber types, innervated by distinct motor neurons, can synchronize with each other and integrate as a network across distant back and leg muscles during squat movements, as revealed by the complex inter-muscular networks of muscle fiber interactions obtained from our empirical analyses.

The uncovered hierarchical reorganization of different-type muscle LegR-BackR and LegR-BackL sub-networks in response to fatigue (Figures 4, 6) reflects a compensatory mechanism to maintain coordination between legs and trunk during squat movements, when the same-type muscle LegL-LegR and BackL-BackR sub-network are fatigued. Remarkably, with repeated exercise this compensatory mechanism is expressed by increased degree of synchronization and coupling of muscle fiber activity within the different-type muscle sub-networks, while at the same time the coupling strength and interactions among muscle fibers within the same-type muscle sub-networks decreases with accumulation of fatigue (Figures 3, 5). Considering that accumulation of fatigue leads to a common effect of reduced motor neuron excitability (lower neuronal discharge rate) in each individual leg or back muscle, it remains an open question what mechanisms could lead to the markedly different response to increased levels of fatigue we uncover for the different-type muscle sub-networks compared to the same-type muscle sub-networks. Notably, the degree of coupling among muscle fibers in the LegL-LegR and BackL-BackR sub-networks is already at maximum level during Exercise 1 (Figures 3C, 5) due to the major role these sub-networks play in generating squat movements that require maximum effort, and thus, the only pathway to respond to fatigue is through a decline in the synchronization and coupling of muscle fiber activity in these sub-networks. In contrast, network links strength of all modules in the Leg-Back sub-networks is at intermediate level during Exercise 1 (Figures 4B, 6), which allows for flexibility to increase the degree of synchronization and coupling among muscle fibers with repeated exercise, and thus, to compensate reduced coordination in the same-type muscle sub-networks with higher levels of fatigue. In this context, our study provides first evidence that fatigue affects not only the function and dynamics of individual muscles but also the ways muscle fibers across muscles synchronize activity and integrate as a network to adapt to fatigue, and that this adaptation process through hierarchical reorganization in network interactions strongly depends on the particular pair of muscles and their role in executing a given movement.

The reported empirical findings indicate that our method (Methods) is sensitive to reveal a hierarchically structured network organization of muscle fiber interactions across muscles with distinct function, histochemical characteristics and effort contribution during movements, and to capture how sub-networks and modules within the global inter-muscular network reorganize with transition from one state to the other (rest vs. exercise) and adapt in response to fatigue. We uncover very complex links strength organization and stratification for the same-type muscle sub-networks (primary motor muscle pairs) compared to the different-type muscle sub-networks (compensatory muscle pairs). Each network module in the same-type muscle sub-networks is characterized by a particular profile of links strength, where with accumulation of fatigue with repeated exercise bouts, all profiles exhibit significant 1) global decline in links strength and 2) increase in links strength stratification (spread), while 3) preserving the general functional form (shape) of each profile (Figure 5). In contrast, all modules in the sub-networks of different-type muscles are characterized by links strength profile of the same shape, and with accumulation of fatigue all profiles exhibit significant 1) global increase in links strength and 2) decline in links strength stratification (spread), while 3) changing the functional form (shape) of each profile (Figure 6). Further, our approach can distinguish between same-type muscle sub-networks with different role in the generation and coordination of movements—while links strength stratification (spread) within each module of the LegL-LegR sub-network dramatically increases with fatigue, the stratification of links strength for the modules of the BackL-BackR sub-network remains unchanged (Figure 7).

## 5 Conclusion

**FIGURE 8:**
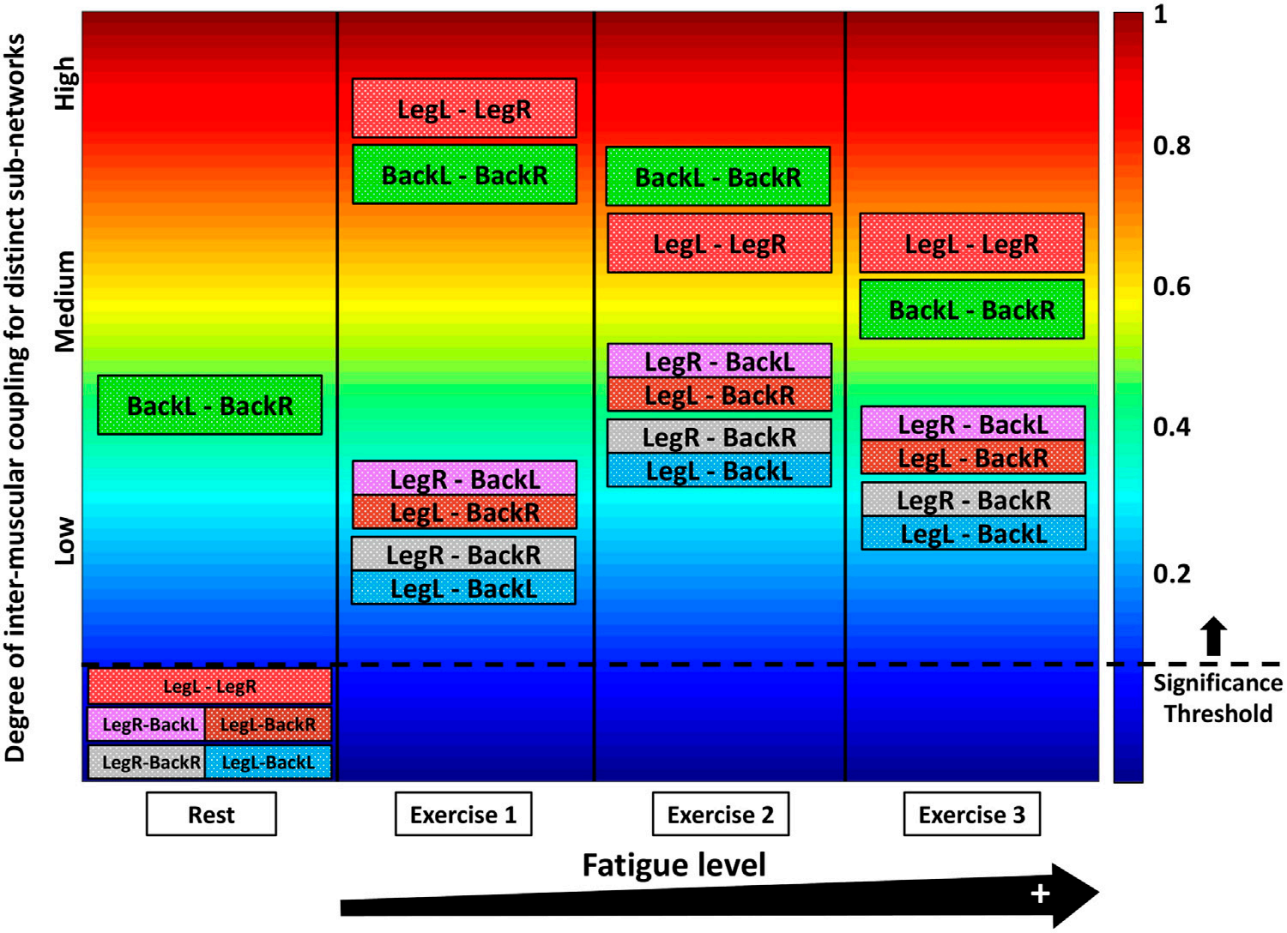
Schematic diagram of the degree of inter-muscular coupling for the major sub-networks involved in rest and squat movement, and evolution in time of network interactions with accumulation of fatigue. At supine Rest, the average network links strength (degree of coupling/coordination of activity among muscle fibers) in all inter-muscular sub-networks is below the significance threshold (marked as horizontal dashed line) except for the BackL-BackR sub-network (Figure 2A), where higher muscle tone and a medium degree of coupling among muscle fibers from the erector spinae left and right muscles relates to the postural role these muscles play under supine resting conditions, compared to the vastus lateralis left and right leg muscles which are not active during supine rest. The average links strength for all major sub-networks (i.e., degree of inter-muscular coupling) significantly increases with transition to squat exercise (Exercise 1), where the same-type muscle sub-networks LegL-LegR and BackL-BackR exhibit high degree of coupling compared to the low and intermediate degree of coupling observed for the different-type muscle LegR-BackR and LegR-BackL sub-networks. This reflects distinct link strength profiles of sub-network interactions that play role in coordinating muscle fibers activity between different-type muscle groups (Figure 6) compared to the same-type muscle sub-networks (Figure 5). With accumulating fatigue during Exercise 2, interactions in the LegL-LegR sub-network become weaker as this sub-network accounts for the major dynamic effort in generating squat movement, and is faster affected by fatigue due to the large fraction of type-II fast muscle fibers involved in this sub-network. In contrast, in the BackL-BackR sub-network links strength remains unchanged during Exercise 2 (same degree of inter-muscular coupling as during Exercise 1) due to the supportive secondary role Back muscles play in squat movement and their predominant composition of fatigue-resistant type-I slow muscle fibers. Notably, the sub-networks composed of different-type muscles (LegR-BackL, LegL-BackR, LegR-BackR and LegL-BackL) play compensatory role in squat movement and elevate the degree of inter-muscular coupling during Exercise 2 to offset fatigue effects in the LegL-LegR sub-network. With further accumulation of fatigue during Exercise 3, the average strength of links in the BackL-BackR sub-network as well as in the four different-type muscles sub-networks (LegR-BackL, LegL-BackR, LegR-BackR and LegL-BackL) significantly declines, although the average links strength in the different-type muscle sub-networks remains higher compared to Exercise 1, indicating that these sub-networks continue to play compensatory role in squat movement even at the level of extreme exhaustion during Exercise 3. Remarkably, there is no decline in the average links strength of the LegL-LegR sub-network during Exercise 3, indicating that (for this particular movement) a bottom level of inter-muscular coupling between LegL and LegR muscle fibers is reached.

In summary, this work addresses inter-muscular interactions among rhythms of myoelectrical activation, corresponding to the activity of different type muscle fibers, across muscles with distinct composition and functions. We report empirical evidence that muscle fibers of different types dynamically synchronize their activity across muscle groups following distinct patterns of cross-frequency communication that depend on muscle characteristics and on the role of muscles in a movement. We demonstrate that muscle fibers integrate as a network with complex hierarchical organization comprising distinct sub-networks and network modules to facilitate coordinated movements. We establish how the global inter-muscular network of muscle fiber interactions reorganizes with transition from rest to exercise (Figure 2A), and with accumulation of fatigue during consecutive exercise bouts (Figure 3A). We discover that each pair of muscles in the global inter-muscular network forms a distinct sub-network (depending on the structure and function of the muscles involved) that is characterized by specific signature of cross-frequency communication among muscle fiber types. Further, each sub-network comprises multiple modules, where each module exhibits a unique profiles of links strength stratification reflecting the degree of synchronization among muscle fibers (Figures 3, 4). Our analyses reveal different organization in sub-networks for pairs of same-type muscles vs. different-type muscles depending on the role of the muscles in the movement. We uncover very complex links strength organization and stratification for the same-type muscle sub-networks (primary motor muscle pairs) compared to the different-type muscle sub-networks (compensatory muscle pairs). Our findings demonstrate a complex dynamic process of coordination among network modules and sub-networks within the global network of muscle fiber interactions, where different network elements 1) adjust their characteristics to facilitate global function of the entire network (Figure 7), and 2) follow distinct phase-space trajectories depending on their specific role in generating physiological states and adapting to levels of fatigue (Figure 8).

The Network Physiology framework (Ivanov, 2021) we employ to investigate cross-frequency coupling of different type muscle fibers across muscle groups during rest and maximal exercise, uncovers physiologically relevant information and leads to new insights on the mechanisms regulating inter-muscular interactions. The reported empirical observations elucidate previously unrecognized aspects of muscle physiology related to basic laws of synchronous muscle fiber activation and network integration to facilitate optimal coordination among muscles to perform a broad range of movements, and adaptation to changes in physiological state and levels of fatigue. The uncovered dynamic network characteristics of muscle fiber interactions can have broad implications for diverse exercise-related phenomena, including sports performance, fatigue, overtraining or muscle-skeletal injuries. From a practical point of view, our approach can be utilized to develop novel network-based biomarkers able to assess and quantify inter-muscular interactions during exercise and movement. Given that each muscle pair presents specific signatures of cross-frequency communication to synchronize activation among distinct muscle fiber types, tracking changes on inter-muscular interaction profiles alongside other physiological markers could help quantify more precisely physiological adaptations after training, and may assist coaches with selection of the most appropriate training programs. For instance, lack of change in the coupling between given type of muscle fibers after a training period, could indicate that previous training intervention was not effective to generate coordinative adaptations in those muscle fibers. This might be of key importance for training programs targeting specific muscle fibers in elderly subjects (Kienbacher et al., 2014) or cancer patients (Thomas, 2022). Further, the developed here dynamic networks approach to muscle fiber inter-muscular interactions could be applied to understand how these interactions change under the pathophysiology of movement disturbances such as freezing of gait in Parkinson’s Disease (Günther et al., 2019) and other neuro-degenerative and neuro-muscular disorders, leading to new biomarkers of disease. More research is needed to 1) confirm the universality of our results over larger cohorts of subjects and identify the reference dynamic network profiles for inter-muscular communication in different age groups; 2) study inter-muscular interactions under various clinical conditions (e.g., acute and chronic muscle injuries, neuro-muscular and neuro-degenerative disorders, etc.), and 3) investigate synchronized cross-frequency communication among different muscles and key organ systems, such as the brain and related cortical rhythms (Ciria et al., 2019; Rizzo et al., 2020, 2022). Extending investigations in these directions would lay down the first building blocks of a new interdisciplinary area of research, Network Physiology of Exercise (Balagué et al., 2020, Balagué et al., 2022a,b).

## Data Availability

The raw data supporting the conclusions of this article will be made available by the authors, without undue reservation.
